# The role of Fe(III) and water in the oxidation of chalcopyrite

**DOI:** 10.1007/s00894-026-06747-y

**Published:** 2026-05-07

**Authors:** Selma Fabiana Bazan, Hélio Anderson Duarte, Guilherme Ferreira de Lima

**Affiliations:** Grupo de Pesquisa Em Química Inorgânica Teórica, Departamento de Química – ICEx, Universidade Federal de Minas Gerais, Av. Antônio Carlos Pampulha, Belo Horizonte, MG 6627 Brazil

**Keywords:** Chalcopyrite, Surface oxidation, Hydrogen transfer, DFT, Leaching kinetics

## Abstract

**Context:**

Chalcopyrite is the most abundant copper sulfide mineral and is becoming increasingly important for meeting the growing demand for copper. However, its slow dissolution during the leaching process remains a technological challenge, in which the key step is the oxidation of chalcopyrite. The initial stages of chalcopyrite oxidation by Fe^3^⁺(aq) were investigated on the sulfur-terminated (001) and (112) surfaces. Adsorption of the [Fe(OH)_3_(H_2_O)_2_] complex is stronger on the (001)-S surface (−18.8 kcal mol⁻^1^) than on (112)-S (−13.4 kcal mol⁻^1^), forming bidentate bonds that promote electron transfer from the surface to the oxidizing agent. The subsequent redox step proceeds through a hydrogen-transfer mechanism, in which water undergoes homolytic dissociation to yield Fe^2^⁺–OH_2_ and an ∙OH radical that attacks surface sulfur atoms, forming S–OH species. This process is both thermodynamically and kinetically favorable on (001)-S (Δ*E* = −6 kcal mol⁻^1^; *E*_*a*_ = 11 kcal mol⁻^1^) but strongly hindered on (112)-S (*E*_*a*_ ≈ 80 kcal mol⁻^1^). The high stabilization of reaction products on (112)-S likely promotes surface passivation, which may explain the kinetic limitations observed experimentally during chalcopyrite leaching. Comparatively, although O_2_ is a stronger oxidant, its low solubility in aqueous media limits its effectiveness relative to Fe^3^⁺.

**Methods:**

Density functional theory (DFT) calculations were performed under periodic boundary conditions using the PW91 exchange–correlation functional and plane-wave basis sets and Vanderbilt ultrasoft pseudopotentials. A Hubbard U correction of 2 eV was applied to surface Fe atoms. Calculations were carried out using the Quantum ESPRESSO 6.2.1 package. Activation barriers were computed using the nudged elastic band method, and atomic charges were obtained through Löwdin population analysis.

**Graphical Abstract:**

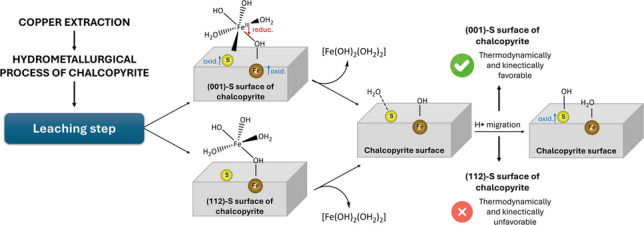

**Supplementary Information:**

The online version contains supplementary material available at 10.1007/s00894-026-06747-y.

## Introduction

Copper is a critical element whose global demand is projected to grow substantially in the coming decades, increasing the risk of a supply crisis. As high-grade copper deposits are depleted, low-grade ores are becoming increasingly important to meet this demand [1–4]. Therefore, considerable efforts are being directed toward the copper extraction from low-grade chalcopyrite ores, despite the significant technological challenges involved.

Chalcopyrite (CuFeS_2_), the most abundant copper-bearing mineral [5], has a tetragonal structure (space group *I4̅2d*) with lattice parameters *a* = *b* = 5.289 Å and *c* = 10.453 Å [6]. It is a semiconductor with a band gap of approximately ~0.5 eV at room temperature and exhibits antiferromagnetic behavior along the *c*-axis [7–10]. The absence of a defined cleavage plane complicates the identification of the predominant surfaces during leaching. However, XPS analyses suggest that multiple surface terminations may coexist, including sulfur-rich (–S) and metal-rich (–M, where M = Cu and/or Fe) surfaces [9–11].

De Lima et al*.* investigated the adsorption of water molecules on metal-rich surfaces and demonstrated that these surfaces exhibit a certain hydrophobic character and lower reactivity toward adsorption [12]*.* Therefore, the present study on the chalcopyrite oxidation considers only the sulfur-rich surfaces.

Hydrometallurgical copper extraction from chalcopyrite typically occurs in acidic media (pH ≈ 2), producing oxides, hydroxides, and sulfides. However, the refractory nature of chalcopyrite leads to a low recovery rate. After a few hours, leaching rates decrease sharply, even in the presence of oxidizing agents such as Fe^3^⁺ [13–17]. Alternative approaches, such as bioleaching with *Acidithiobacillus ferrooxidans* [18], offer environmentally friendly routes, but the fundamental surface oxidation mechanisms remain unclear.

Experimental studies have identified various sulfur species on leached chalcopyrite surfaces, including elemental sulfur and sulfates, whose formation is promoted in the presence of O_2_ [9, 19]. Previous theoretical and experimental studies on H_2_O and O_2_ adsorption on (001) and (112) chalcopyrite surfaces revealed preferential interaction with Fe atoms, hydrogen bonding to surface S atoms, and possible homolytic cleavage of O_2_ [12, 20]. Other work has shown strong Fe^3^⁺ adsorption on sulfur-rich surfaces (−305 kcal mol^−1^); however, these studies modeled the leaching agent as a bare ion, neglecting aqueous hydrolysis and hydration effects [21]. The dramatic difference relative to the hydrated complex (−18.8 kcal mol⁻^1^, this work) underscores the importance of explicitly including ion solvation in the surface model.

In earlier work, we modeled the adsorption of the hydrated complex [Fe(OH)_3_(H_2_O)_2_] on (001)-S and (112)-S chalcopyrite surfaces, demonstrating preferential adsorption on the (001)-S surface through bidentate coordination involving Fe_ads_–S_surf_ and O_ads_–Fe_surf_ bonds. This adsorption promoted oxidation of surface Fe and S atoms, suggesting a potential initial step in the leaching process [22].

We have revisited the initial steps of the chalcopyrite oxidation by [Fe(OH)_3_(H_2_O)_2_] on the (001)-S and (112)-S terminations, specifically investigating the role of adsorbed water in promoting sulfur atoms oxidation. According to Santos et al. [23], pyrite oxidation occurs via type I and type II hydrogen-transfer mechanisms on the surface. Type I reactions involve oxidation of two Fe(II) sites to form predominantly the Fe(III)-OH^−^. In chalcopyrite, this is formed by ∙OH transfer from the oxidizing [Fe(OH)_3_(H_2_O)_2_] leaching agent to the surface Fe(II) sites. Type II reactions exhibit higher activation energies and lead to S-OH bond formation through the hydrogen atom transfer from a water molecule to the Fe(III)–OH^–^ species, yielding Fe(II)–OH_2_. By analogy with pyrite oxidation—where hydrogen transfer between adsorbed H_2_O and ∙OH is critical—we hypothesize that a similar process occurs in chalcopyrite, with its feasibility depending on surface termination [23–26]. We investigated the electronic structure of the species, reaction energies, and barriers to elucidate reactivity differences that may explain the kinetic limitations of chalcopyrite leaching compared to pyrite.

## Methodology

We followed the same computational protocol as in our previous work [22], employing density functional theory (DFT) within the generalized gradient approximation (GGA) with the PW91 exchange–correlation functional and plane-wave basis sets [27]. The PW91 functional accurately reproduces the structure and electronic properties of chalcopyrite and has been widely employed in related computational studies [12, 22, 28, 29]. Its performance is closely comparable to PBE for this class of materials.

Vanderbilt ultrasoft pseudopotentials were used to describe core electrons, while the valence electrons of Fe (3s^2^3p^6^3d^6.5^4s^1^), Cu (3d^9.5^4s^1^4p^0.5^), S (3s^2^3p^4^), O (2s^2^2p^4^), and H (1s^1^) were expanded in plane waves. The Hubbard *U* parameter of 2 eV was applied to surface Fe atoms, which exhibit partially filled electronic states. This value was chosen based on previous DFT+U studies of chalcopyrite [6, 7, 22, 30] and because it provides the best agreement with both the experimental band gap (~0.5 eV) and the reported structural parameters, as validated in the “Results and discussion” section. All calculations were performed using the Quantum ESPRESSO 6.2.1 package [31–33].

The sulfur-terminated (001) and stepped (112) surfaces were generated from the optimized bulk structure, with the bottom three layers fixed to their bulk positions. For the (001) surface, a 2 × 2 slab containing 64 atoms was created using a 16 Å vacuum layer, 40 Ry kinetic energy cutoff for the plane waves, and a 2 × 2 × 1 Monkhorst–Pack *k*-point mesh [34], which was later refined to 4 × 4 × 2 for electronic structure analyses. For the (112) surface, a 2 × 2 slab containing 128 atoms was generated with a 20 Å vacuum layer and a 30 Ry kinetic energy cutoff. Initial sampling used the Γ-point, which was subsequently refined to 2 × 2 × 1 for electronic structure analyses. These protocols were selected to ensure total energy convergence within 10⁻^3^ Ry/atom for each slab geometry, consistent with the protocol established in our previous work [22].

Magnetic ordering followed the most stable antiferromagnetic arrangement reported by Conejeros et al., with alternating Fe spin layers along the slab direction [35]. Atomic charges were evaluated via Löwdin analysis. Activation barriers were computed using the nudged elastic band (NEB) method [36–38].

The adsorption energy, $\Delta {E}_{ads}$, was calculated using Eq. (1).

1 $$\Delta {E}_{ads}={E}_{surface+molecule}-{E}_{surface}-{E}_{molecule}$$

where ${E}_{surface}$ is the total energy of the pristine surface, ${E}_{molecule}$ is the total energy of the isolated molecule placed in the same unit cell using the same protocol, and ${E}_{surface+molecule}$ is the total energy of the adsorbed system.

## Results and discussion

The optimized lattice parameters, *a* = *b* = 5.276 Å and c = 10.464 Å, are in close agreement with the experimental values of *a* = *b* = 5.289 Å and *c* = 10.453 Å [6]. The band gap was estimated to be 0.5 eV, which matches the experimental value well [7]. The density of states (DOS) is shown in Figure S1 of the Supplementary information.

**Fig. 1:**
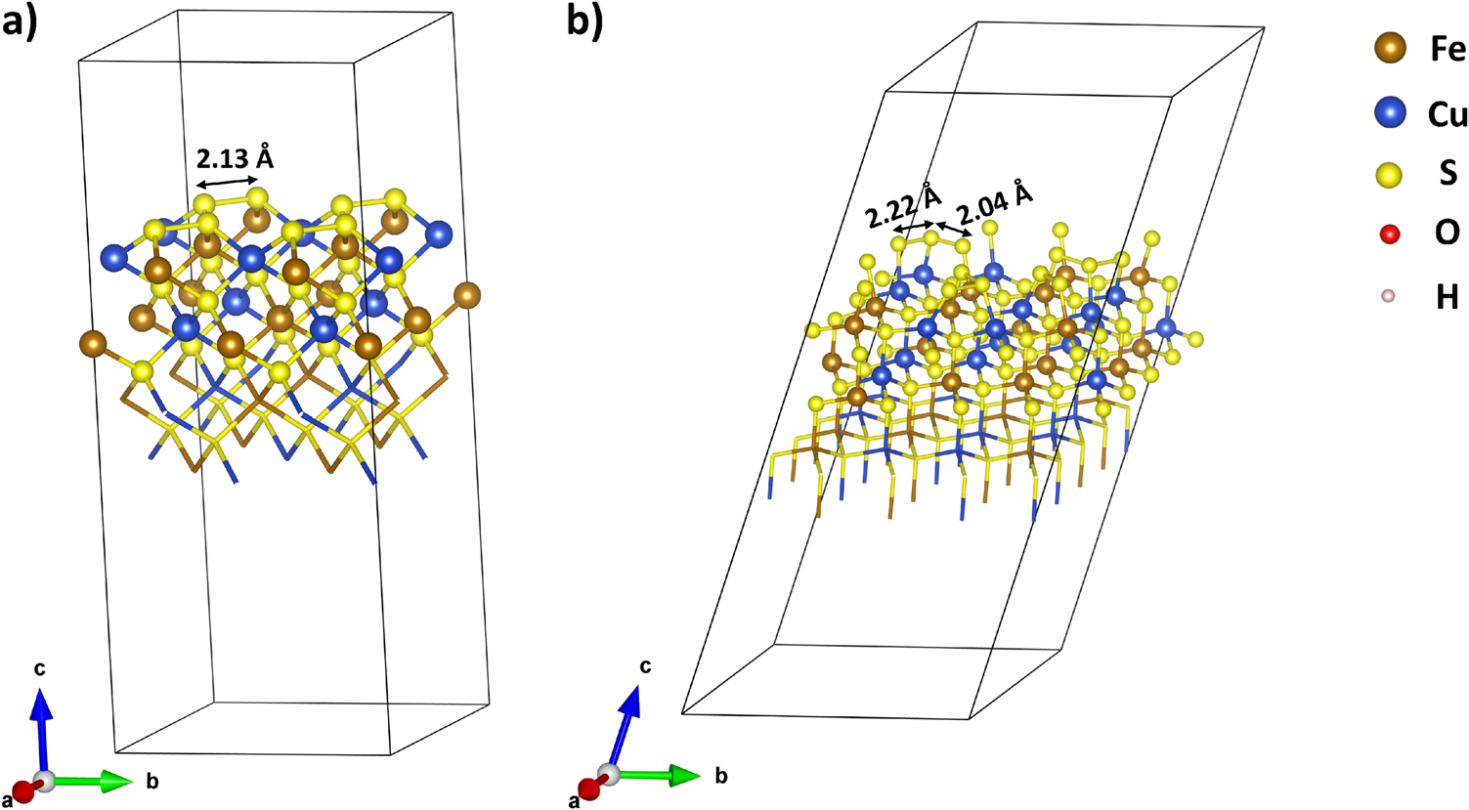
Optimized structure of the (**a**) (001)-S and (**b**) (112)-S chalcopyrite surfaces. The disulfide species ${S}_{2}^{2-}$ formed upon surface relaxation are highlighted

Figure 1 illustrates the structure of the (001)-S and (112)-S surfaces, each consisting of eight atomic layers.

Surface optimization led to the formation of disulfide dimers, reproducing the results reported by Duarte and co-authors [28, 29]. Different DFT studies [39, 40] have shown that bulk chalcopyrite is consistent with an Fe^3+^Cu^+^S_2_^2−^ structure. Surface reconstruction was investigated in detail by de Oliveira et al. [29], who showed that the formation of surface sulfur dimers leads to the oxidation of the surface S^2−^ to S_2_^2−^ species, followed by the reduction of the surface Fe^3+^ to Fe^2+^. The presence of leaching agents promotes further oxidation of the S_2_^2−^ species. The S–S bond distance on relaxed (001)-S and (112)-S surfaces is predicted to be 2.13 and 2.04 Å, respectively. These values compare to 2.12 and 2.26 Å for (001)-S obtained by de Oliveira et al. [29]. The S_2_^2^⁻ dimers on both surfaces are clearly visible in Fig. 1, where they appear as pairs of surface sulfur atoms with shorter-than-bulk S–S distances. Experimental evidence of the formation of the S_2_^2−^ on chalcopyrite surfaces has also been reported [41, 42].

Chalcopyrite leaching has been shown to release Fe^2+^ species from the surface, accompanied by the formation of sulfates and acid, as revealed in Eq. (2) [16].

2 $${\mathrm{CuFeS}}_{2}+16{\mathrm{Fe}}^{3+}+8{\mathrm{H}}_{2}\text{O }\to \text{ C}{\mathrm{u}}^{2+}+17{\mathrm{Fe}}^{2+}+2{\mathrm{SO}}_{4}^{2-}+16{\mathrm{H}}^{+}$$

The oxidation process releasing Fe^2+^ and SO_4_^2−^ species is not fully understood, as it is generally considered that leaching agents such as Fe^3+^(aq) oxidize surface Fe^2+^. However, the results of de Oliveira et al. [29] suggest that the formation of surface Fe^2^⁺ sites, promoted by the generation of S_2_^2^⁻ dimers, leads to the population of the conduction band, which possesses predominant antibonding character. This electronic redistribution facilitates the release of Fe^2^⁺ species into solution.

The Fe^3+^ species in aqueous solution were modeled as sextet bipyramidal [Fe(OH)_3_(H_2_O)_2_] complexes, as proposed by de Abreu et al. [43] in their DFT study of Fe^3+^(aq) hydrolysis. Using the neutral [Fe(OH)_3_(H_2_O)_2_] species as a reference is essential to avoid introducing artificial charges into the periodic system. We investigated the adsorption of [Fe(OH)_3_(H_2_O)_2_] following the approach of Bazan et al. [22], who examined various adsorption sites on the (001)-S and (112)-S surfaces using DFT calculations. On the (001)-S surface, the [Fe(OH)_3_(H_2_O)_2_] complex interacts with surface Fe^2+^ sites through its OH groups and with sulfur atoms via the Fe center. In contrast, on the (112)-S surface, [Fe(OH)_3_(H_2_O)_2_] interacts with Fe^2+^ sites exclusively through the OH groups.

**Fig. 2:**
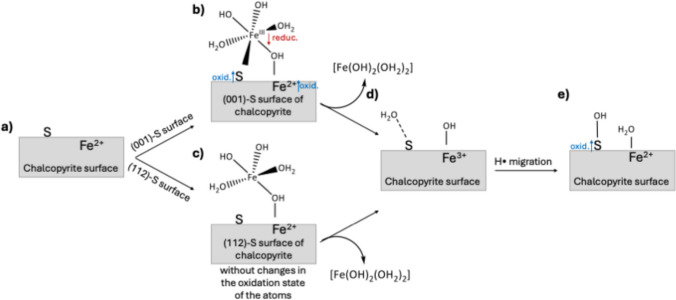
Proposed mechanism of the surface oxidation of chalcopyrite by Fe^3^⁺(aq) assisted by adsorbed water. (**a**) Clean chalcopyrite surface prior to reaction. Adsorption of the [Fe(OH)_3_(H_2_O)_2_] oxidizing agent on the (**b**) (001)-S surface via bidentate coordination (S_surf_–Fe_ads_ and O_ads_–Fe_surf_ bonds) and on the (**c**) (112)-S surface via monodentate coordination (O_ads_–Fe_surf_ bond only). (**d**) Intermediate state after ∙OH transfer from the leaching agent to a surface Fe(II) site, yielding a surface Fe(III)–OH⁻ species. (**e**) Type II hydrogen-transfer step: a water molecule undergoes homolytic dissociation, transferring ∙H to Fe(III)–OH⁻ to produce Fe(II)–OH_2_, while the resulting ∙OH radical attacks a surface sulfur atom to form S–OH. Bond lengths are given in Å

We have revisited these calculations and proposed an oxidation mechanism as depicted in Fig. 2. Adsorption on the (001)-S surface involves S_surf_–Fe_ads_ and O_ads_–Fe_surf_ bonding, with calculated lengths of 2.47 Å and 2.02 Å, respectively, as shown in Fig. 2b. The estimated adsorption energy for this bidentate mode is −18.8 kcal mol^−1^. In contrast, adsorption on the (112)-S surface occurs via monodentate mode, forming O_ads_–Fe_surf_ bond with a length of 2.04 Å (Fig. 2c). The adsorption energy is −13.4 kcal mol^−1^, which is approximately 5 kcal mol^−1^ less favorable than on the (001)-S surface. These results are in excellent agreement with our previous calculations [22]. Figure S2 in the Supplementary Information shows the geometries of the optimized structures.

Bazan et al. performed a detailed analysis of Bader charges and projected density of states (PDOS), revealing that the adsorbed Fe(III) species undergoes reduction to Fe(II), accompanied by the oxidation of the chalcopyrite surface [22]. We calculated the PDOS for the first two layers of the (001)-S surface before and after the adsorption indicating that the surface undergoes oxidation (see Figure S3). Adsorption represents the initial step in chalcopyrite oxidation by the Fe^3^⁺ leaching agent, wherein surface Fe(II) sites are subsequently oxidized to form Fe(III) species. This oxidized intermediate, represented in Fig. 2d, precedes the hydrogen-transfer step.

Dos Santos et al. demonstrated that the type II reaction on the pyrite (FeS_2_) surface occurs via hydrogen transfer from adsorbed water molecules to form Fe(II)–OH_2_, while the sulfur atom is attacked by the resulting ∙OH radical [23]. We hypothesized that these two reaction steps are also involved in the chalcopyrite oxidation mechanism in the presence of water, as illustrated in Fig. 2d and e.

Therefore, we propose that upon desorption of the Fe^2^⁺ species (the reduced leaching agent), a hydroxyl radical (∙OH) is transferred to adjacent surface Fe(II) sites, promoting their oxidation to Fe^3^⁺–OH⁻ and sustaining the redox cycle of chalcopyrite dissolution.

According to Guimarães et al., the neutral Fe^2+^ species in aqueous solution is a triplet quasi-planar *trans*-[Fe(OH)_2_(OH_2_)_2_] species [44]. The [Fe(OH)_2_(OH_2_)_2_] species requires 32.2 kcal mol⁻^1^ to leave the (001)-S surface and 78.3 kcal mol⁻^1^ to leave the (112)-S surface, indicating that this process is less favorable on (112)-S. Water is preferentially adsorbed on the Fe centers through a molecular pathway with an estimated adsorption energy of −10.8 kcal mol^−1^. Table 1 shows the adsorption energy of the leaching agents O_2_ and [Fe(OH)_3_(H_2_O)_2_] and their respective reduced species, H_2_O and [Fe(OH)_2_(H_2_O)_2_]. Interestingly, O_2_ adsorbs more strongly (≈ −70 kcal mol^−1^) than the Fe^3+^ species (≈ −15 kcal mol^−1^). The reduced species formed from the redox reaction on the surface exhibit significantly lower adsorption energies, indicating that the surface remains available for continued oxidation in the presence of further leaching agents.

**Table 1:** Adsorption energy (Δ*E*_ads_) of the processes with the leaching agents and their respective coordination modes on the (001)-S and (112)-S surfaces of chalcopyrite

Process	Δ*E*_ads_ (kcal mol^−1^)	
	(001)-S	(112)-S
Surface + [Fe(OH)_3_(H_2_O)_2_] → surface-[Fe(OH)_3_(H_2_O)_2_]	−18.8 Bidentate (S, Fe)	−13.4 Monodentate (Fe)
Surface + O_2_ → surface-O_2_^a^	−76.5 Bidentate (S, Fe)	−64.2 Bidentate (S, Fe)
Surface + H_2_O → surface-H_2_O	−9.7 (−22.8)^b^ Monodentate (Fe)	−2.6 Monodentate (Fe)

^a^Ref [22]. ^b^Ref [12]

**Fig. 3:**
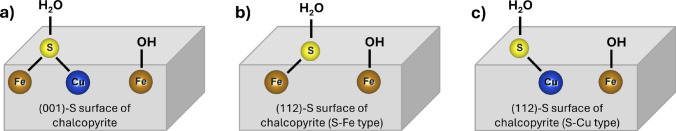
Adsorption models of H_2_O and ∙OH species on the surface. **a** (001)-S, **b** (112)-S of the S-Fe type, and (**c**) (112)-S of the S-Cu type

The type II reaction can occur in the presence of water, involving the transfer of a hydrogen atom (∙H) from water to Fe(III)–OH⁻ to form Fe(II)–OH_2_, while the resulting hydroxyl radical (·OH) attacks surface sulfur atoms, promoting their oxidation. On the (001)-S surface, the symmetric arrangement of surface Fe and S atoms defines a single migration pathway, illustrated in Fig. 3a. In contrast, the (112)-S surface contains two non-equivalent sulfur atoms, resulting in two possible migration modes: one involving sulfur bound to iron (S–Fe, Fig. 3b) and one involving sulfur bound to copper (S–Cu, Fig. 3c).

**Fig. 4:**
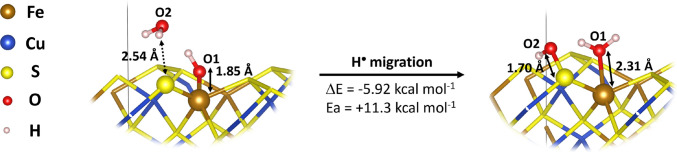
Optimized structures of the hydrogen-transfer process (mechanism II) on the chalcopyrite (001)-S surface. The diagram illustrates the homolytic dissociation of a water molecule, with the simultaneous migration of the ∙H radical to the Fe(III)–OH⁻ site (forming Fe(II)–OH_2_) and the attack of the resulting ∙OH radical on the surface sulfur atom to form the S–OH species. Reaction energies (Δ*E*), activation energies (*Eₐ*), bond lengths (solid lines in Å), and intermolecular interactions (dashed lines) are presented. For an extended view of the entire surface, please refer to Figure S4 in the Supplementary Information

For the (001)-S surface, the reaction is exothermic (Δ*E* = −5.9 kcal mol⁻^1^) with a calculated energy barrier of 11.3 kcal mol⁻^1^ (Fig. 4 and Table 2). Structural optimizations revealed that, in all cases, the water molecule does not bind directly to sulfur, corroborating the surface’s hydrophobic nature. The hydrogen migration from water to Fe(III)–OH⁻, which forms Fe(II)–OH_2_, occurs concomitantly with the attack of the ∙OH radical on the sulfur atom. The reactant and product of this process are shown in Fig. 4, along with the corresponding bond lengths and intermolecular distances.

**Table 2:** Adsorption energies (Δ*E*_ads_) and activation energies (*E*_*a*_), both in kcal mol⁻^1^, for hydrogen migration on the (001)-S, (112)-S-Fe, and (112)-S-Cu chalcopyrite surfaces

	Δ*E*_ads_	*E*_*a*_
(001)-S	−5.92	+11.3
(112)-S-Fe	+13.86	+79.4
(112)-S-Cu	+5.97	+83.3

**Fig. 5:**
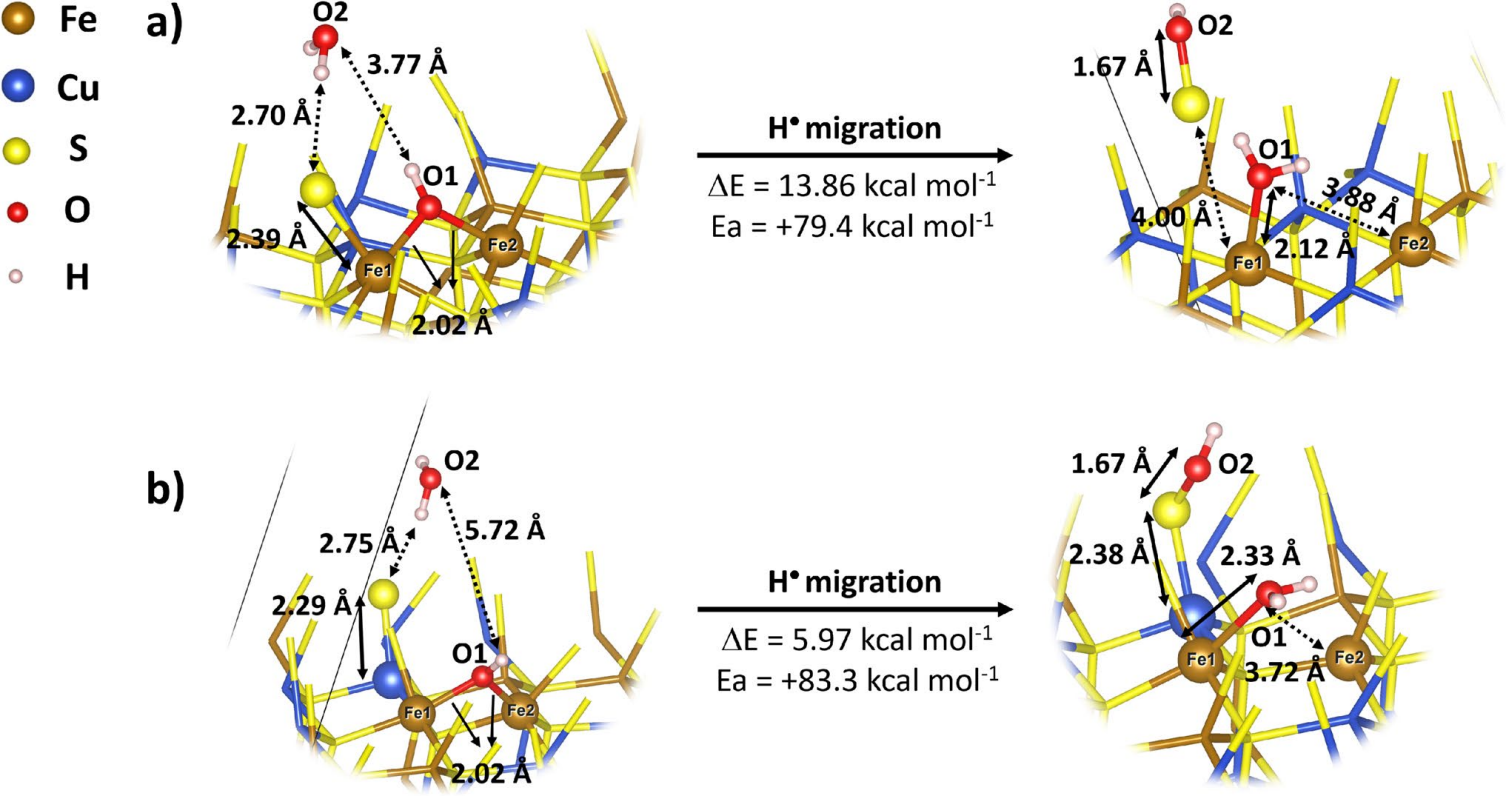
Optimized structures of the hydrogen transfer on the chalcopyrite (112)-S surface for two distinct site models: **a** sulfur bonded to iron (S–Fe) and (**b**) sulfur bonded to copper (S–Cu). The calculated energy barriers for both pathways (~80 kcal mol⁻^1^) are significantly higher than those of the (001)-S face, evidencing the kinetic limitation of this surface termination. The diagram details the reaction energies (Δ*E*), activation energies (*Eₐ*), interatomic distances (Å), and the hydrogen interactions involved in the transition state. This figure focuses on the reaction sites. For an extended view of the entire surface, please refer to Figure S5 in the Supplementary Information

Figure 5 and Table 2 show the two pathways for the ∙H transfer on the (112)-S surface. The reaction is endothermic for both pathways (Δ*E* = 13.9 and 6.0 kcal mol^−1^), with energy barriers of approximately 79.4 and 83.3 kcal mol^−1^ for S–Fe and S–Cu models, respectively (Fig. 5a and b, respectively). These barriers are roughly eight times higher than those calculated for the (001)-S surface. The presence of Cu or Fe does not appreciably interfere with the energy barrier or reaction energy. The difference in Δ*E* and energy barriers appears to be related to the structure of the adsorption site, as the iron and the sulfur centers are more accessible on the (001)-S surface. Furthermore, the (001)-S surface presents lower energy barriers, which are comparable to the type II mechanism of pyrite oxidation, where the energy barrier is estimated to be approximately 11–19 kcal mol^−1^ [23].

**Fig. 6:**
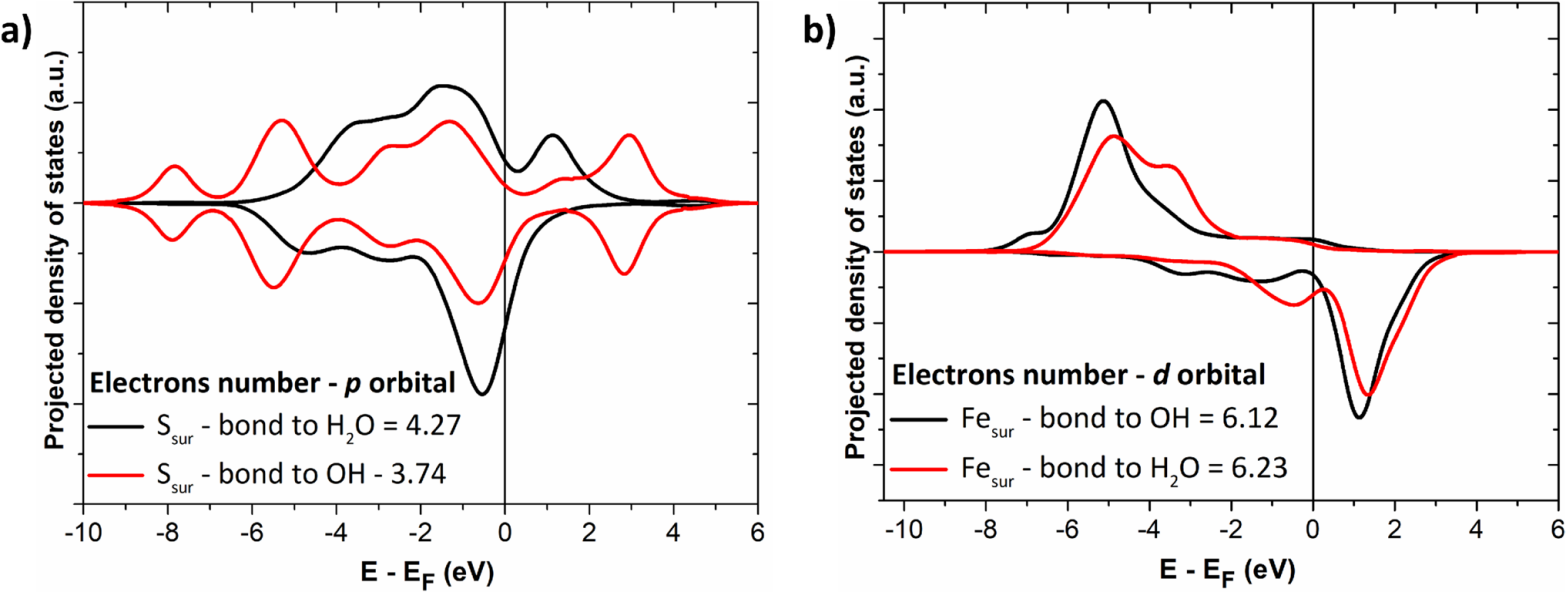
Projected density of states (PDOS) for the (**a**) 3*p* orbitals of the sulfur atom and (**b**) 3*d* orbitals of the iron atom on the (001)-S chalcopyrite surface

Projected density of states (PDOS) analysis for the (001)-S surface (Fig. 6) revealed that sulfur atoms are oxidized during hydrogen transfer, while Fe atoms tend to be reduced. As shown in Fig. 6a, the sulfur 3p states shift to lower energies upon hydrogen transfer, indicating oxidation. Conversely, Fig. 6b shows a redistribution of the iron 3 d states toward the Fermi level, consistent with the partial reduction of surface Fe. Löwdin charge analysis confirmed these trends: the sulfur atom involved in the migration showed a charge increase of +0.46 *e*, and the oxygen atom involved showed a decrease of 0.11 e, consistent with ∙H transfer.

The endothermicity of the reactions on the (112)-S surface (approximately 6 kcal mol⁻^1^) does not entirely preclude their occurrence. However, the energy barriers of approximately 80 kcal mol⁻^1^ are excessively high, likely preventing the reaction from proceeding due to unfavorable kinetics. Furthermore, the strong adsorption energy of [Fe(OH)_2_(H_2_O)_2_], −78.3 kcal mol^−1^, may contribute to surface passivation, hindering the approach of further leaching agents. The disparity in surface reactivity could explain the experimentally observed decrease in leaching rates over time as more reactive surfaces are consumed.

## Conclusion

The extraction of copper from low-grade ores via hydrometallurgical processes remains challenging due to the hindered dissolution of chalcopyrite, which results in remarkably slow leaching kinetics. This behavior contrasts with that of other sulfides in the pyrite group. In the early stages of pyrite oxidation under oxidative and humid conditions, Fe(III) species are generated. The presence of Fe(III) accelerates the oxidation of sulfide minerals, such as pyrite, leading to the release of Fe^2^⁺, sulfate, and acid. The acid produced further dissolves surface reaction products, exposing fresh mineral surfaces to the environment. The Fe^3^⁺—an oxidative derivative of the Fe^2^⁺ released from the surface—then recycles and sustains the overall oxidation process.

The initial step of the chalcopyrite oxidation mechanism in the presence of the Fe(III) leaching agent was investigated. Fe(III) was modeled as the neutral [Fe(OH)_3_(H_2_O)_2_] complex to avoid artifacts in the periodic calculations arising from charged species. The redox product was represented by the neutral [Fe(OH)_2_(H_2_O)_2_] complex.

Adsorption of [Fe(OH)_3_(H_2_O)_2_] on the surface is followed by oxidation via the transfer of an ∙OH species to surface Fe(II) sites, forming Fe(III)–OH⁻ species. A type II reaction mechanism was identified, in which a water molecule undergoes homolytic dissociation, transferring an ∙H to Fe(III)–OH⁻ to yield Fe(II)–OH_2_, while the resulting ∙OH radical attacks surface sulfur atoms to form S–OH groups. This mechanism is both thermodynamically and kinetically favorable on the (001)-S surface, with a reaction energy of −6 kcal mol⁻^1^ and an activation barrier of 11 kcal mol⁻^1^. In contrast, on the (112)-S surface, the energy barrier is considerably higher, preventing the reaction. Moreover, the reaction products are significantly more stabilized on this surface (Δ*E*_*ads*_ = −78.3 kcal mol⁻^1^, corresponding to a desorption cost of +78.3 kcal mol⁻^1^), contributing to its passivation and hindering the access of leaching agents. Our results indicate that the (001)-S termination of chalcopyrite exhibits a higher oxidative reactivity.

Molecular oxygen (O_2_) acts as a much more effective leaching agent, and it is readily adsorbed and dissociatively activated, promoting surface oxidation. In acidic aqueous media, this reaction leads to the formation of H_2_O, a weakly adsorbed species that is easily desorbed, with an adsorption energy less negative than −10 kcal mol⁻^1^. Despite the intrinsically higher effectiveness of O_2_ as an oxidizing agent, its availability at the mineral–water interface is limited by its low solubility compared to Fe^3^⁺. Although we have not exhaustively explored all chalcopyrite surface terminations, our results indicate that different surfaces can exhibit markedly distinct chemical reactivities, which may account for the kinetic limitations commonly observed during chalcopyrite leaching.

## Supplementary Information

Below is the link to the electronic supplementary material.

ESM 1 Supplementary Material 1 (DOCX 2.61 MB)

## Data Availability

No datasets were generated or analysed during the current study.
