# Supplementary material for: The role of Fe(III) and water in the oxidation of chalcopyrite
Source: J Mol Model. 2026 May 7;32(6):165. doi: 10.1007/s00894-026-06747-y (PMC13153022; doi:10.1007/s00894-026-06747-y)
Supplement: Supplementary file 1 — Supplementary Material 1 (DOCX 2.61 MB) [file 894_2026_6747_MOESM1_ESM.docx]

**Supplementary Information**

**for**

**The Role of Fe(III) and Water in the Oxidation of Chalcopyrite**

Selma Fabiana Bazan¹, Hélio Anderson Duarte¹, and Guilherme Ferreira de Lima¹*

¹ Universidade Federal de Minas Gerais, Grupo de Pesquisa em Química Inorgânica Teórica, Departamento de Química – ICEx, Av. Antônio Carlos, 6627, Pampulha, Belo Horizonte – MG, Brazil, 31270–901.

*Corresponding author: gflima@ufmg.br

**Table of contents**

[Results 3](#_Toc214741375)

[Cartesians coordinate (in Å) of optimized structures 8](#_Toc214741376)

Results





**Figure S1.** Density of States (DOS) of bulk chalcopyrite.


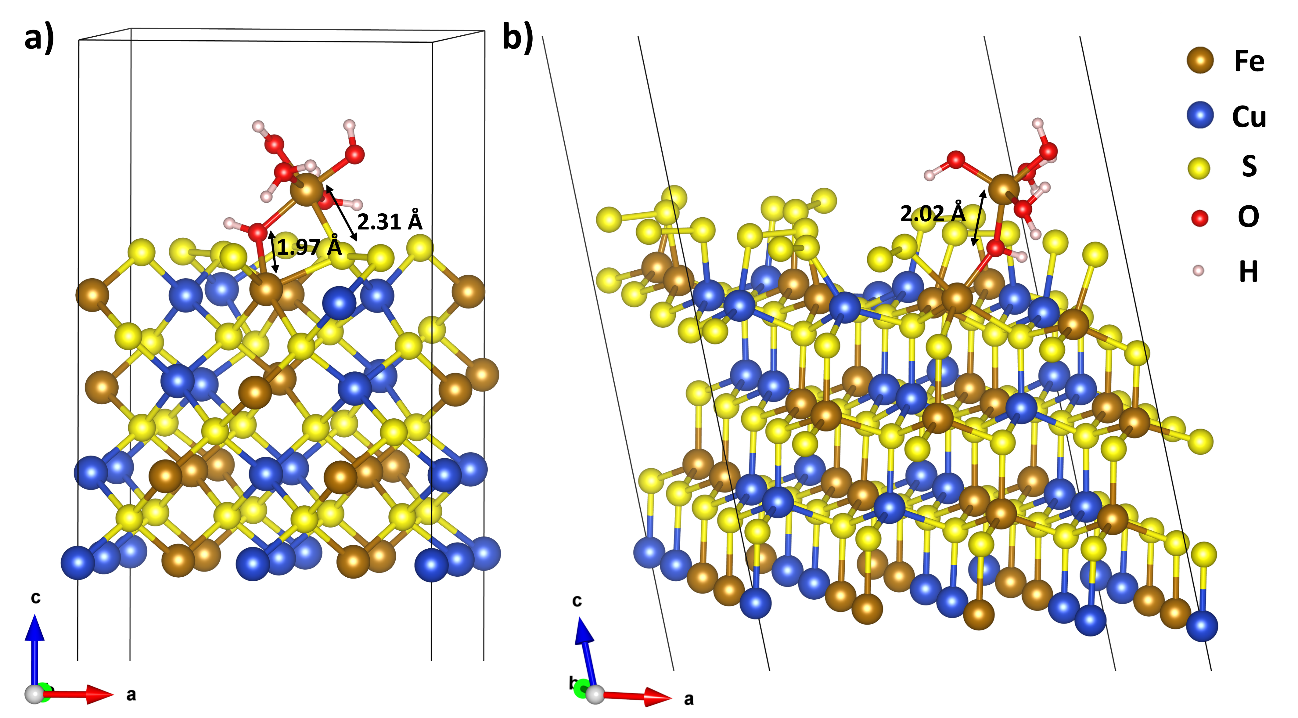


**Figure S2.** Optimized structures of the adsorption of the hydrated iron ion on the a) (001)-S and b) (112)-S surfaces of chalcopyrite.





**Figure S3.** PDOS for the first two layers of the (001)-S surface before and after the adsorption


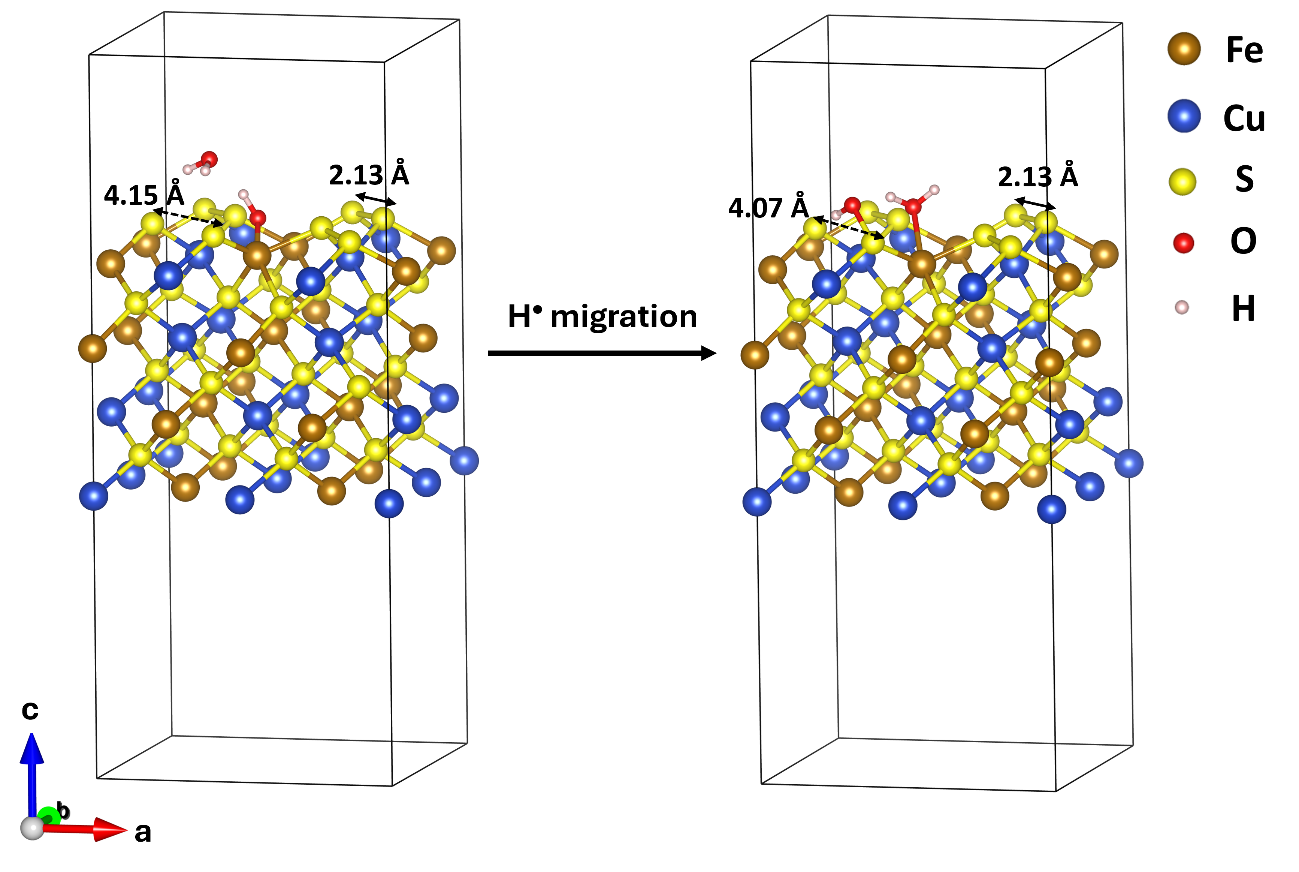


**Figure S4.** Optimized structures of the hydrogen migration on the (001)-S surface of chalcopyrite.


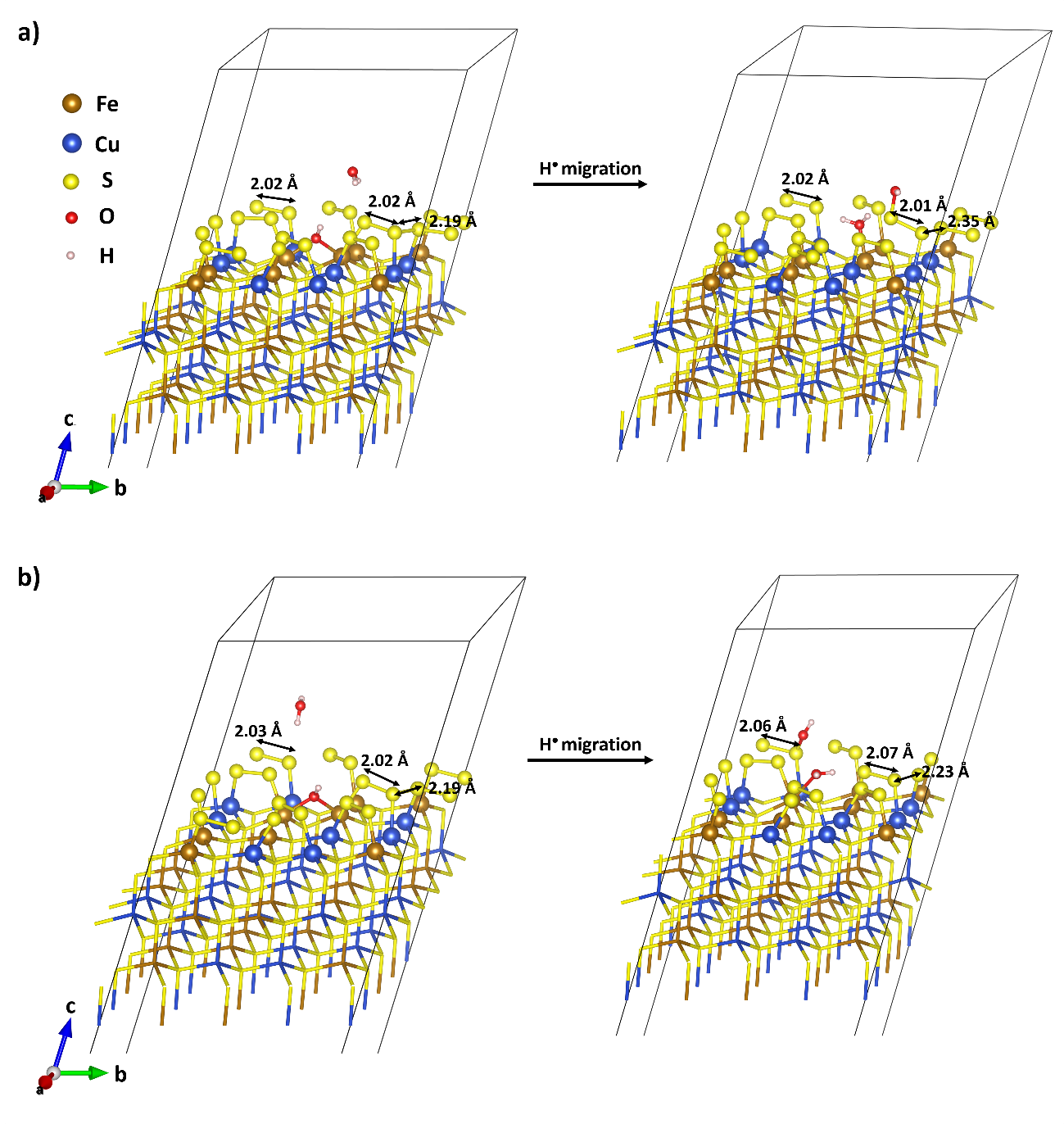


**Figure S5.** Optimized structures of the hydrogen transfer on the chalcopyrite (112)-S surface for two distinct site models: (a) sulfur bonded to iron (S–Fe) and (b) sulfur bonded to copper (S–Cu).

Cartesians coordinate (in Å) of optimized structures

(001)-S surface of chalcopyrite

S 3.370180549 1.879375033 19.027953509

S 8.718301115 1.875169289 19.014829992

S 1.878330655 3.404322817 19.025019652

S 7.230618671 3.403343089 19.018567305

S 3.366449618 7.227580806 19.014870265

S 8.718188874 7.227071651 19.004246509

S 1.879607915 8.755911505 19.008632944

S 7.229364459 8.753135987 18.999667136

Cu 2.630448629 -0.023142585 17.857280762

Cu 7.980248161 -0.028569981 17.842836569

Fe2 -0.043935732 2.640104288 17.874801003

Fe2 5.300025124 2.637275576 17.885413752

Cu 2.618497046 5.315853712 17.868235269

Cu 7.972497345 5.311816618 17.855808363

Fe2 -0.045731276 7.991935801 17.860792689

Fe2 5.296772589 7.986730448 17.870558357

S 1.213695149 1.246015439 16.516742649

S 6.559720593 1.248014070 16.519063831

S 4.033809877 4.031728001 16.538656510

S 9.379971815 4.028468935 16.517581594

S 1.214207681 6.592281538 16.515253376

S 6.561873585 6.592952066 16.519168524

S 4.032619125 9.376409531 16.515511380

S 9.379476865 9.374786895 16.495616515

Fe1 -0.040523539 -0.032224417 15.130248051

Fe1 5.305554732 -0.040903480 15.148395088

Cu 2.629298289 2.641303816 15.302307614

Cu 7.980421902 2.626222017 15.285899475

Fe1 -0.051153568 5.312221000 15.146606751

Fe1 5.295064409 5.304748023 15.165065885

Cu 2.618118801 7.989702582 15.281601565

Cu 7.969651955 7.975048266 15.268310573

S 3.951801700 1.325082301 13.919972094

S 9.294367597 1.322925760 13.883426135

S 1.320185435 3.965542649 13.914697264

S 6.643377352 3.950064380 13.923352789

S 3.932933951 6.642624285 13.920568883

S 9.276343484 6.640670206 13.885589346

S 1.319079459 9.310904730 13.879608699

S 6.642831890 9.294043858 13.889742529

Fe2 2.644500000 0.000000000 12.605750000

Fe2 7.933500000 0.000000000 12.605750000

Cu 0.000000000 2.644500000 12.605750000

Cu 5.289000000 2.644500000 12.605750000

Fe2 2.644500000 5.289000000 12.605750000

Fe2 7.933500000 5.289000000 12.605750000

Cu 0.000000000 7.933500000 12.605750000

Cu 5.289000000 7.933500000 12.605750000

S 1.361389000 1.322250000 11.302875000

S 6.650389000 1.322250000 11.302875000

S 3.927611000 3.966750000 11.302875000

S 9.216611000 3.966750000 11.302875000

S 1.361389000 6.611250000 11.302875000

S 6.650389000 6.611250000 11.302875000

S 3.927611000 9.255750000 11.302875000

S 9.216611000 9.255750000 11.302875000

Cu 0.000000000 0.000000000 10.000000000

Cu 5.289000000 0.000000000 10.000000000

Fe1 2.644500000 2.644500000 10.000000000

Fe1 7.933500000 2.644500000 10.000000000

Cu 0.000000000 5.289000000 10.000000000

Cu 5.289000000 5.289000000 10.000000000

Fe1 2.644500000 7.933500000 10.000000000

Fe1 7.933500000 7.933500000 10.000000000

---------

(112)-S surface of chalcopyrite

S -1.919332415 4.331651674 21.328372957

S -3.849941050 5.427602141 21.344703226

S -7.278456597 7.428568571 21.361634800

S 4.715570868 9.239405257 21.317848859

S 2.796294671 10.364787335 21.340851376

S -0.673903694 12.332204215 21.374737288

S -3.656282422 13.955289888 21.323258742

S -5.588021449 15.063829313 21.345274677

S 2.364896764 4.081182756 21.412025391

S 6.973911370 5.502293696 21.400227593

S -3.144958024 7.344927611 21.392775591

S 1.466291141 8.813022320 21.401360764

S -8.723970303 10.716216014 21.411492549

S -4.271192650 12.007247819 21.421232022

S 0.690256481 13.818708942 21.404780984

S -9.723201452 15.279645978 21.453157375

Cu -4.606025430 4.944609174 19.157482323

Cu -1.046442067 4.850025530 19.167401820

Fe2 2.701431744 4.804434177 19.241979283

Fe1 6.537046444 4.937613619 19.246527855

Cu -6.595394768 8.031852930 19.280954437

Fe2 -2.860379774 8.036438199 19.215515248

Fe1 0.969988712 8.230842570 19.229820215

Cu 4.660025651 8.220754867 19.156247854

Fe2 -8.383984695 11.279618187 19.213974959

Fe1 -4.589744797 11.414778277 19.228856220

Cu -0.880289943 11.453423256 19.289340904

Cu 2.803233832 11.254910249 19.152082750

Fe1 -10.092918086 14.674959710 19.259435689

Cu -6.346751498 14.629891005 19.156733975

Cu -2.816605438 14.491426691 19.153188742

Fe2 0.946310518 14.466850183 19.245598054

S 4.595166912 6.015744594 18.576818471

S 0.828748814 5.998794554 18.568304830

S -0.992040922 9.272624866 18.570416773

S -4.750165687 9.195479157 18.557451899

S -6.512744024 12.458223474 18.508818466

S -10.227531422 12.468494666 18.522106638

S -12.056205789 15.678869372 18.545359190

S -0.933893357 15.640809296 18.564830204

S -6.478659839 5.924398776 18.318294874

S -2.780048005 5.997415483 18.219560652

S 2.786546497 9.145269453 18.221541674

S -8.310979739 9.197386004 18.287641714

S -2.740123764 12.372307623 18.293195750

S 1.011800933 12.385855052 18.319522687

S -8.217586682 15.639541592 18.354470770

S -4.526580263 15.696451628 18.228875319

Cu -4.720702916 2.746757572 16.230282275

Fe2 -0.961034008 2.727307406 16.093701549

Fe1 2.764965107 2.749227428 15.996638409

Cu 6.423186193 2.752517549 16.244312425

Fe1 -2.780498919 5.975981722 15.987990609

Cu 0.872244453 5.988471827 16.252380412

Cu 4.592359332 5.986057295 16.258385455

Fe2 -6.508732836 5.943652503 16.066659294

Cu -4.677993115 9.215600509 16.244891378

Cu -0.965436528 9.222765273 16.255557112

Fe2 2.794707809 9.168148812 15.990369746

Fe1 -8.323067959 9.193422835 16.043504095

Cu -10.203597144 12.454452881 16.214131883

Cu -6.496525455 12.450720272 16.202124294

Fe2 -2.734808815 12.399218275 16.046637801

Fe1 0.981180657 12.421516864 16.068284343

S 0.872108848 3.879460369 15.321571687

S -2.871857514 3.778499222 15.325528237

S -4.674457001 7.105793174 15.317620187

S 6.433185230 7.014484627 15.325510653

S 4.656755945 10.333510590 15.317835696

S 0.874274373 10.253341389 15.319961685

S -0.887328660 13.561273850 15.327150460

S -4.640830596 13.479791969 15.317277215

S 4.622816336 3.861683370 15.324124634

S 8.283127399 3.791866203 15.333725368

S -0.923703859 7.097067405 15.324968146

S 2.742032040 7.000417517 15.324872682

S -6.480372679 10.313055692 15.323456547

S -2.811559354 10.243133503 15.330901469

S 2.821088752 13.537753816 15.329367698

S -8.342237331 13.505158641 15.329362468

Cu -2.864590000 3.834704000 13.038470000

Cu 0.847998000 3.834704000 13.038470000

Fe2 4.560587000 3.834704000 13.038470000

Fe1 8.273175000 3.834704000 13.038470000

Cu -4.693485000 7.065564000 13.038470000

Fe2 -0.980896000 7.065564000 13.038470000

Fe1 2.731692000 7.065564000 13.038470000

Cu 6.444281000 7.065564000 13.038470000

Fe2 -6.522380000 10.296424000 13.038470000

Fe1 -2.809791000 10.296424000 13.038470000

Cu 0.902797000 10.296424000 13.038470000

Cu 4.615386000 10.296424000 13.038470000

Fe1 -8.351274000 13.527284000 13.038470000

Cu -4.638686000 13.527284000 13.038470000

Cu -0.926097000 13.527284000 13.038470000

Fe2 2.786491000 13.527284000 13.038470000

S 6.416881000 4.884947000 12.301337000

S 2.732171000 4.932763000 12.301337000

S 0.875398000 8.115807000 12.301337000

S -2.809312000 8.163624000 12.301337000

S -4.666085000 11.346667000 12.301337000

S -8.350795000 11.394484000 12.301337000

S -10.207568000 14.577527000 12.301337000

S 0.958076000 14.625344000 12.301337000

S -4.748763000 4.901201000 12.256368000

S -1.008296000 4.949017000 12.256368000

S 4.560108000 8.132061000 12.256368000

S -6.549779000 8.179878000 12.256368000

S -0.981375000 11.362921000 12.256368000

S 2.759092000 11.410738000 12.256368000

S -6.522859000 14.593781000 12.256368000

S -2.782391000 14.641598000 12.256368000

Cu -2.919390000 1.702097000 10.000000000

Fe2 0.793199000 1.702097000 10.000000000

Fe1 4.505787000 1.702097000 10.000000000

Cu 8.218376000 1.702097000 10.000000000

Fe2 -4.748284000 4.932957000 10.000000000

Fe1 -1.035696000 4.932957000 10.000000000

Cu 2.676893000 4.932957000 10.000000000

Cu 6.389481000 4.932957000 10.000000000

Fe1 -6.577179000 8.163817000 10.000000000

Cu -2.864590000 8.163817000 10.000000000

Cu 0.847998000 8.163817000 10.000000000

Fe2 4.560587000 8.163817000 10.000000000

Cu -8.406073000 11.394677000 10.000000000

Cu -4.693485000 11.394677000 10.000000000

Fe2 -0.980896000 11.394677000 10.000000000

Fe1 2.731692000 11.394677000 10.000000000

---------

Adsorption of Fe^3+^ on (001)-S surface of chalcopyrite

O2 7.8026193884 3.6852317858 22.1340703662

H 7.5740699963 4.1051505406 22.9894681606

O2 5.2465416625 4.5086853577 22.3944074872

H 4.8650807983 3.8014342714 22.9496859882

O 5.9158465835 1.9545410451 21.6493105865

H 6.7783836641 1.5431106010 21.8652134198

O 6.5788909055 5.7393428446 20.6949790478

H 6.2278244675 6.1399672345 21.5207110538

H 5.4678223783 1.4177480636 20.9304313879

H 7.5170276821 6.0376521575 20.5502364763

Fe3 6.3311492625 3.8126618312 21.0674874321

O2 4.8749331603 3.6715615854 19.7759826500

H 3.9767573424 3.9639501785 20.0413956426

S 4.6039624328 0.5410985036 19.1513751773

S 8.8721840325 1.7658306987 19.1094799452

S 1.3254798577 3.9333049195 19.1616590631

S 7.4726132797 3.3443864155 19.1131063775

S 3.3318830085 7.1693143909 19.0009822440

S 9.3423947643 6.6496804089 19.2565275507

S 1.8703515555 8.6874393636 19.0314681432

S 6.1493257395 9.7752148007 19.0328815939

Cu 2.7861950008 -0.2228108518 17.8535165165

Cu 7.8750435526 0.0834810678 17.8097705635

Fe2 0.0184264886 2.7320322642 17.9125285381

Fe2 5.2896483771 2.6257563904 18.1262134128

Cu 2.4623646009 5.3115201651 17.8349881793

Cu 8.2475589181 5.3024561610 17.8650990061

Fe2 -0.0539070578 7.8074195458 17.9205877710

Fe2 5.1326740693 8.0697894569 17.7955969267

S 1.3371186578 1.1981174604 16.7419491413

S 6.5315280412 1.4096806023 16.4597477791

S 3.9612902459 3.9568565586 16.6610045941

S 9.4037071249 3.9391373841 16.4164600829

S 1.1939870353 6.6129921191 16.3882176655

S 6.6240701457 6.6103671109 16.7426751518

S 3.9824513226 9.2930816489 16.2529402624

S 9.3064838431 9.3338789491 16.7222538202

Fe1 -0.0518198660 -0.0426745811 15.3446403416

Fe1 5.3461644164 0.0065789041 15.1230897093

Cu 2.6216341785 2.5849440538 15.3672979736

Cu 7.9710687218 2.6265606030 15.1751220213

Fe1 -0.0696153912 5.2876369750 15.0716502795

Fe1 5.3067152327 5.2970849327 15.3650307027

Cu 2.5536296531 7.9347379718 15.0920765167

Cu 7.9498924068 7.9753156339 15.3747562322

S 3.9419848620 1.3453316183 13.9142054890

S 9.3525155757 1.2671151316 13.9226433555

S 1.3505687020 3.9204408629 13.9197686890

S 6.5897917892 4.0037647399 13.9434378232

S 4.0046822759 6.5735524499 13.9379097101

S 9.2395964066 6.6741280643 13.8972429932

S 1.2315730271 9.3740524501 13.8940222610

S 6.6695129793 9.2741999151 13.9078934065

Fe2 2.6445000000 0.0000000000 12.6057500000

Fe2 7.9335000000 0.0000000000 12.6057500000

Cu 0.0000000000 2.6445000000 12.6057500000

Cu 5.2890000000 2.6445000000 12.6057500000

Fe2 2.6445000000 5.2890000000 12.6057500000

Fe2 7.9335000000 5.2890000000 12.6057500000

Cu 0.0000000000 7.9335000000 12.6057500000

Cu 5.2890000000 7.9335000000 12.6057500000

S 1.3613890000 1.3222500000 11.3028750000

S 6.6503890000 1.3222500000 11.3028750000

S 3.9276110000 3.9667500000 11.3028750000

S 9.2166110000 3.9667500000 11.3028750000

S 1.3613890000 6.6112500000 11.3028750000

S 6.6503890000 6.6112500000 11.3028750000

S 3.9276110000 9.2557500000 11.3028750000

S 9.2166110000 9.2557500000 11.3028750000

Cu 0.0000000000 0.0000000000 10.0000000000

Cu 5.2890000000 0.0000000000 10.0000000000

Fe1 2.6445000000 2.6445000000 10.0000000000

Fe1 7.9335000000 2.6445000000 10.0000000000

Cu 0.0000000000 5.2890000000 10.0000000000

Cu 5.2890000000 5.2890000000 10.0000000000

Fe1 2.6445000000 7.9335000000 10.0000000000

Fe1 7.9335000000 7.9335000000 10.0000000000

---------

Adsorption of Fe^3+^ on (112)-S surface of chalcopyrite

Fe3 4.1352631086 4.8777430693 23.3231265393

O2 3.7910605086 5.2308454539 21.3953659639

H 4.4921438669 5.8435123696 21.0793831635

O 4.2512159984 7.0403072279 23.6816144682

H 4.3743449554 7.7404666607 22.9977314267

O2 5.5610890169 4.9756546946 24.6098241344

H 5.2449418480 4.7714025824 25.5113467353

O 5.3155694011 3.0241337520 22.9352005551

H 5.9415944670 3.3402955581 23.6329443764

O2 2.6770556696 4.3390288698 24.2604325621

H 1.8946145383 4.0458584423 23.7458187659

H 5.8529081558 2.6965811037 22.1821916910

H 5.0857588823 6.9543933162 24.1962091022

S -2.1950934109 4.2492875440 21.2035131174

S -4.6080542434 6.0307625941 21.2800267598

S -6.5301382451 7.2157004872 21.2605399213

S 4.6135147102 9.2497818354 21.2155328248

S 2.6757277591 10.3855697091 21.2965078408

S -0.6761339400 12.3107869537 21.3797131233

S -4.3860566781 13.6666707514 21.6768124514

S -6.3318572830 14.6237540475 21.2338665730

S 1.2182481797 3.6833810616 21.4565843130

S 6.9891234680 5.6502598794 21.2963201836

S -3.2010441893 7.5122038650 21.3767696255

S 1.3894182247 8.8082247492 21.3893624414

S -8.7216659152 10.6044895417 21.3863325785

S -4.7489850454 11.6882149379 21.4986766660

S 0.4823497957 13.9734626341 21.3647529652

S -9.8022804788 15.1787473906 21.3481020336

Cu -4.6153781162 5.0086349876 19.1476926013

Cu -1.0508732294 4.8125687439 19.2807302755

Fe2 2.6827884333 4.7778714564 19.7678562238

Fe1 6.4742870986 5.0774448322 19.0911550946

Cu -6.5235505901 8.0309756756 19.0880544045

Fe2 -2.8517608130 8.0820710298 19.1621471554

Fe1 0.8974184768 8.1739445372 19.2039782923

Cu 4.5761361781 8.1869835071 19.0666559185

Fe2 -8.4482279027 11.2136196586 19.1589922248

Fe1 -4.6802735177 11.3882920013 19.2111627244

Cu -0.9057117716 11.4853891939 19.2957689868

Cu 2.7151541162 11.2546610283 19.1202693834

Fe1 -10.1663495240 14.6260087826 19.1566450245

Cu -6.4789947755 14.4918297644 18.9206622282

Cu -2.7807173271 14.6052329814 18.5723774767

Fe2 0.9076247759 14.5306989470 19.1824296731

S 4.4599461671 5.9833051754 18.5048304360

S 0.8654437794 5.9383419739 18.6787551383

S -0.9995371367 9.2908932964 18.5525956224

S -4.6930432078 9.2315929804 18.4617489534

S -6.5827051976 12.3152864128 18.3884972847

S -10.2728637029 12.4359581716 18.5063059027

S -12.0786345426 15.7242954000 18.5353722019

S -0.8826478799 15.7678257668 18.4926963366

S -6.4828789382 5.9203134359 18.1707119020

S -2.7521441629 6.0082044640 18.2429237391

S 2.6950544058 9.1426909721 18.1986672101

S -8.3426785654 9.1090826419 18.2723642343

S -2.8071988107 12.3978359624 18.3713351805

S 0.9577724728 12.4343226546 18.2969298896

S -8.3248655330 15.6396864868 18.2679175142

S -4.6522333082 15.7688638985 18.3947998314

Cu -4.7316925743 2.7419575494 16.2185853880

Fe2 -1.0046534430 2.7488857845 16.0170241179

Fe1 2.6973521181 2.7898740807 16.1156273321

Cu 6.3990789221 2.7529840542 16.1797747214

Fe1 -2.7858334110 5.9869017391 15.9980133152

Cu 0.8818873297 5.9786351103 16.3407361834

Cu 4.5486204134 5.9603587973 16.2172056836

Fe2 -6.5329510787 5.9542848299 15.9433541588

Cu -4.6532351867 9.2228087102 16.1535906609

Cu -0.9689541797 9.2144418707 16.2294568367

Fe2 2.7420922807 9.1810364740 15.9651381906

Fe1 -8.3785354615 9.1789765245 16.0131778115

Cu -10.2216255151 12.4416036644 16.1933491061

Cu -6.5035213130 12.4327986196 16.0950125996

Fe2 -2.7982812077 12.4429196568 16.0826216963

Fe1 0.9333297165 12.4464223580 16.0384403027

S 0.8516124595 3.8801584409 15.3354494287

S -2.8919618549 3.8154293030 15.3178547514

S -4.6576215430 7.0845017137 15.3054481421

S 6.4038482837 7.0155240606 15.3095078817

S 4.6187751947 10.3069697269 15.3169943919

S 0.8642995075 10.2889371423 15.3143627941

S -0.9380408604 13.5340764427 15.3250162139

S -4.6501613941 13.5505143056 15.3293648262

S 4.5623715460 3.8366623496 15.3215730003

S 8.2736858538 3.7759901719 15.3063311885

S -0.9091780652 7.0827856933 15.3348391557

S 2.7041931106 7.0058399604 15.3181190370

S -6.5101437051 10.2746334121 15.3076276171

S -2.7971075230 10.2921042235 15.3262959686

S 2.7997044267 13.5352554058 15.3250729626

S -8.3722051941 13.5050050614 15.3132048614

Cu -2.8645900000 3.8347040000 13.0384700000

Cu 0.8479980000 3.8347040000 13.0384700000

Fe2 4.5605870000 3.8347040000 13.0384700000

Fe1 8.2731750000 3.8347040000 13.0384700000

Cu -4.6934850000 7.0655640000 13.0384700000

Fe2 -0.9808960000 7.0655640000 13.0384700000

Fe1 2.7316920000 7.0655640000 13.0384700000

Cu 6.4442810000 7.0655640000 13.0384700000

Fe2 -6.5223800000 10.2964240000 13.0384700000

Fe1 -2.8097910000 10.2964240000 13.0384700000

Cu 0.9027970000 10.2964240000 13.0384700000

Cu 4.6153860000 10.2964240000 13.0384700000

Fe1 -8.3512740000 13.5272840000 13.0384700000

Cu -4.6386860000 13.5272840000 13.0384700000

Cu -0.9260970000 13.5272840000 13.0384700000

Fe2 2.7864910000 13.5272840000 13.0384700000

S 6.4168810000 4.8849470000 12.3013370000

S 2.7321710000 4.9327630000 12.3013370000

S 0.8753980000 8.1158070000 12.3013370000

S -2.8093120000 8.1636240000 12.3013370000

S -4.6660850000 11.3466670000 12.3013370000

S -8.3507950000 11.3944840000 12.3013370000

S -10.2075680000 14.5775270000 12.3013370000

S 0.9580760000 14.6253440000 12.3013370000

S -4.7487630000 4.9012010000 12.2563680000

S -1.0082960000 4.9490170000 12.2563680000

S 4.5601080000 8.1320610000 12.2563680000

S -6.5497790000 8.1798780000 12.2563680000

S -0.9813750000 11.3629210000 12.2563680000

S 2.7590920000 11.4107380000 12.2563680000

S -6.5228590000 14.5937810000 12.2563680000

S -2.7823910000 14.6415980000 12.2563680000

Cu -2.9193900000 1.7020970000 10.0000000000

Fe2 0.7931990000 1.7020970000 10.0000000000

Fe1 4.5057870000 1.7020970000 10.0000000000

Cu 8.2183760000 1.7020970000 10.0000000000

Fe2 -4.7482840000 4.9329570000 10.0000000000

Fe1 -1.0356960000 4.9329570000 10.0000000000

Cu 2.6768930000 4.9329570000 10.0000000000

Cu 6.3894810000 4.9329570000 10.0000000000

Fe1 -6.5771790000 8.1638170000 10.0000000000

Cu -2.8645900000 8.1638170000 10.0000000000

Cu 0.8479980000 8.1638170000 10.0000000000

Fe2 4.5605870000 8.1638170000 10.0000000000

Cu -8.4060730000 11.3946770000 10.0000000000

Cu -4.6934850000 11.3946770000 10.0000000000

Fe2 -0.9808960000 11.3946770000 10.0000000000

Fe1 2.7316920000 11.3946770000 10.0000000000

---------

Structure of the (001)-S surface of chalcopyrite with H_2_O and •OH before H• migration

O 4.966165902 4.227944057 19.362640169

H 4.513649578 3.983510701 20.224211512

O 3.449360096 3.380034527 21.520815308

H 2.599226185 3.645254815 21.103237314

H 3.541800614 2.439570693 21.252758649

S 4.136683877 1.141274346 19.154648578

S 8.898110010 1.783577277 18.964414770

S 1.191694966 4.069197702 19.093430727

S 7.500380400 3.309631279 19.206961952

S 3.381053922 7.209780576 18.982065436

S 8.730118876 7.211554361 19.011779712

S 1.893858105 8.707246786 18.953140575

S 7.238441094 8.729381994 18.981874670

Cu 2.815995666 -0.103848978 17.886616285

Cu 7.948902331 -0.047102980 17.805439264

Fe2 -0.006305686 2.792825595 17.827447164

Fe2 5.296388630 2.776225571 18.258479813

Cu 2.563213672 5.323899779 17.850909848

Cu 8.017717092 5.252121358 17.918486156

Fe2 -0.040963937 7.950750541 17.823641966

Fe2 5.321029598 7.944747501 17.844431065

S 1.300325100 1.241848744 16.684452926

S 6.519237119 1.368791439 16.648054296

S 3.946961354 3.933843637 16.577694327

S 9.328961469 4.026887099 16.432635766

S 1.211016991 6.625042487 16.414788796

S 6.585644620 6.517141343 16.537322598

S 4.094240044 9.300750857 16.425316729

S 9.389155364 9.370395023 16.492016748

Fe1 0.012014189 0.023975601 15.219788739

Fe1 5.369709163 0.017517481 15.204919979

Cu 2.606412525 2.576419053 15.313180313

Cu 7.922199554 2.584715698 15.273075099

Fe1 -0.065635322 5.311545021 15.103319235

Fe1 5.240393838 5.227678709 15.246650970

Cu 2.629909179 7.989816289 15.202080981

Cu 7.952324649 7.961157200 15.284537804

S 3.967342596 1.306137995 13.927131972

S 9.308715393 1.309404117 13.913051007

S 1.330694430 3.945243935 13.917009316

S 6.620058864 3.957352761 13.927381076

S 3.967864353 6.604583128 13.923093782

S 9.260407529 6.656248710 13.872227700

S 1.296991950 9.345931045 13.888090968

S 6.645121285 9.308004160 13.906961260

Fe2 2.644500000 0.000000000 12.605750000

Fe2 7.933500000 0.000000000 12.605750000

Cu 0.000000000 2.644500000 12.605750000

Cu 5.289000000 2.644500000 12.605750000

Fe2 2.644500000 5.289000000 12.605750000

Fe2 7.933500000 5.289000000 12.605750000

Cu 0.000000000 7.933500000 12.605750000

Cu 5.289000000 7.933500000 12.605750000

S 1.361389000 1.322250000 11.302875000

S 6.650389000 1.322250000 11.302875000

S 3.927611000 3.966750000 11.302875000

S 9.216611000 3.966750000 11.302875000

S 1.361389000 6.611250000 11.302875000

S 6.650389000 6.611250000 11.302875000

S 3.927611000 9.255750000 11.302875000

S 9.216611000 9.255750000 11.302875000

Cu 0.000000000 0.000000000 10.000000000

Cu 5.289000000 0.000000000 10.000000000

Fe1 2.644500000 2.644500000 10.000000000

Fe1 7.933500000 2.644500000 10.000000000

Cu 0.000000000 5.289000000 10.000000000

Cu 5.289000000 5.289000000 10.000000000

Fe1 2.644500000 7.933500000 10.000000000

Fe1 7.933500000 7.933500000 10.000000000

---------

Structure of the (001)-S surface of chalcopyrite with H_2_O and •OH after H• migration

O 4.768060811 3.988177160 19.970402089

H 4.118755513 3.361823681 20.396041036

O 3.117881121 1.893440164 20.305000138

H 2.376668889 2.421285091 19.851527862

H 5.528324635 4.053043601 20.582447019

S 4.050095330 1.099063913 19.119155314

S 8.861468422 1.783537736 18.978829642

S 1.190202360 3.992486211 19.118277238

S 7.505090554 3.372983736 19.115809528

S 3.362900780 7.207437365 18.991737558

S 8.722276265 7.207038349 19.029588433

S 1.854126818 8.694556163 18.988576643

S 7.225002339 8.726492114 19.001752640

Cu 2.681880415 -0.170249095 17.815260983

Cu 7.954182306 -0.067177015 17.820970764

Fe2 -0.039484452 2.758409049 17.809188541

Fe2 5.390547819 2.710669118 18.153064522

Cu 2.490172869 5.345058910 17.884363276

Cu 8.058669186 5.295746908 17.842011304

Fe2 -0.059405709 7.946451206 17.834328716

Fe2 5.305043844 7.950173955 17.856280808

S 1.282386211 1.254743099 16.641423604

S 6.540551541 1.293884955 16.599177311

S 3.979382335 3.974641389 16.706714488

S 9.339836464 4.015670276 16.395551453

S 1.191948799 6.626257140 16.418240703

S 6.580733788 6.543833098 16.529741173

S 4.075710859 9.293402417 16.432400835

S 9.385400339 9.365665139 16.493634496

Fe1 -0.000387178 0.007487499 15.202161629

Fe1 5.337499458 -0.004345317 15.179938716

Cu 2.659867771 2.631134841 15.362380073

Cu 7.921490022 2.575852046 15.250164091

Fe1 -0.055866212 5.312600815 15.081652130

Fe1 5.238049891 5.225617782 15.266348096

Cu 2.621142480 7.974688386 15.203057302

Cu 7.954362699 7.971891748 15.276345952

S 3.952544599 1.325121504 13.935445094

S 9.313005219 1.304119625 13.898597840

S 1.349365193 3.937380555 13.922392632

S 6.612149039 3.961301416 13.933424650

S 3.970685729 6.596521921 13.928424278

S 9.259028773 6.658103313 13.868086274

S 1.299799004 9.339271206 13.883583533

S 6.642496645 9.304480148 13.896088519

Fe2 2.644500000 0.000000000 12.605750000

Fe2 7.933500000 0.000000000 12.605750000

Cu 0.000000000 2.644500000 12.605750000

Cu 5.289000000 2.644500000 12.605750000

Fe2 2.644500000 5.289000000 12.605750000

Fe2 7.933500000 5.289000000 12.605750000

Cu 0.000000000 7.933500000 12.605750000

Cu 5.289000000 7.933500000 12.605750000

S 1.361389000 1.322250000 11.302875000

S 6.650389000 1.322250000 11.302875000

S 3.927611000 3.966750000 11.302875000

S 9.216611000 3.966750000 11.302875000

S 1.361389000 6.611250000 11.302875000

S 6.650389000 6.611250000 11.302875000

S 3.927611000 9.255750000 11.302875000

S 9.216611000 9.255750000 11.302875000

Cu 0.000000000 0.000000000 10.000000000

Cu 5.289000000 0.000000000 10.000000000

Fe1 2.644500000 2.644500000 10.000000000

Fe1 7.933500000 2.644500000 10.000000000

Cu 0.000000000 5.289000000 10.000000000

Cu 5.289000000 5.289000000 10.000000000

Fe1 2.644500000 7.933500000 10.000000000

Fe1 7.933500000 7.933500000 10.000000000

---------

Structure of the (112)-S surface of chalcopyrite (S-Fe type) with H_2_O and •OH before H• migration

O -3.396505379 9.833388408 20.680576057

H -2.844150014 10.132881457 21.429042590

O -2.727829601 11.830039182 24.796747620

H -1.941022455 12.155846962 24.322461949

H -3.464121117 12.019194939 24.183562406

S -1.946782761 4.267743453 21.332322745

S -3.972433410 5.176787798 21.341592023

S -7.486039745 7.452194024 21.269844690

S 4.705031711 9.231971268 21.315139906

S 2.780882399 10.371228626 21.326292364

S -0.619632706 12.294623430 21.374272911

S -3.753876670 14.259789655 21.362958546

S -5.688321381 15.284765181 21.336806772

S 2.158417927 4.326177373 21.347772075

S 6.922049955 5.485901461 21.380828704

S -3.614969835 7.145052657 21.539852266

S 1.453458583 8.813930574 21.375781295

S -8.734945728 10.709452898 21.414694495

S -4.413202051 12.372463917 21.677170614

S 0.696098534 13.825426882 21.402412873

S -9.659049682 15.192203044 21.438157903

Cu -4.622072008 4.889708185 19.097030495

Cu -1.136955294 4.882618862 19.154917035

Fe2 2.633327434 4.885336998 19.164169487

Fe2 6.498096214 4.914380216 19.226383733

Cu -6.669173009 8.002344384 19.211023083

Fe2 -3.047129503 8.244884723 19.485139364

Fe2 0.920902434 8.263613836 19.204709560

Cu 4.634754048 8.224367002 19.157590735

Fe2 -8.340004972 11.300883317 19.228126578

Fe2 -4.438294124 11.184783916 19.605687667

Cu -0.880705515 11.457953994 19.260775489

Cu 2.810993580 11.255767685 19.138092151

Fe2 -10.085962094 14.649571921 19.245384466

Cu -6.374978254 14.604917432 19.136253633

Cu -2.880629390 14.538945188 19.162201875

Fe2 0.930629191 14.450933631 19.236096945

S 4.573949170 6.028454223 18.554365177

S 0.739798837 6.040412385 18.519804977

S -1.050593853 9.262619719 18.556070681

S -4.890642871 9.155279774 18.377081278

S -6.370944422 12.379786154 18.654506150

S -10.204430809 12.449656103 18.511888365

S -12.059103028 15.629470848 18.520120731

S -0.958444932 15.619303988 18.561424756

S -6.505640656 5.882670786 18.301463074

S -2.850456273 6.182470346 18.368770943

S 2.761252092 9.144302724 18.211295886

S -8.367117341 9.236471361 18.262250589

S -2.811255050 12.382548973 18.417292896

S 1.012064481 12.377264595 18.307251589

S -8.236871691 15.663958510 18.353007936

S -4.579133157 15.739058769 18.225138898

Cu -4.716595553 2.747335967 16.207153789

Fe1 -0.983027126 2.731976350 16.091002516

Fe1 2.743204226 2.754558413 15.992070322

Cu 6.404192182 2.734657107 16.241304106

Fe1 -2.803370518 6.011018155 16.117071970

Cu 0.837410693 6.005441158 16.215175836

Cu 4.579313201 5.983824064 16.239397383

Fe1 -6.530927798 5.924800252 16.049991753

Cu -4.722477523 9.216877459 16.114515911

Cu -0.998497618 9.228112887 16.254275352

Fe1 2.763324068 9.168789980 15.981447283

Fe1 -8.361846758 9.202267295 16.023803518

Cu -10.193569262 12.465126462 16.202055678

Cu -6.476958200 12.456979925 16.323009453

Fe1 -2.773588326 12.407246478 16.155156354

Fe1 0.963380625 12.426325329 16.057304888

S 0.850489928 3.884903471 15.315773538

S -2.878006410 3.828117212 15.347958103

S -4.687656305 7.076778800 15.307109228

S 6.421329831 7.017840400 15.322047550

S 4.618771533 10.342962266 15.312043990

S 0.843727585 10.255509694 15.317489325

S -0.929969230 13.539588742 15.341884179

S -4.654665252 13.478051854 15.336162141

S 4.610279306 3.855658272 15.321830682

S 8.265936106 3.771240631 15.329860758

S -0.973985030 7.107012505 15.327089150

S 2.726549703 7.001308646 15.318936919

S -6.538645593 10.353334223 15.313417747

S -2.835407847 10.279172895 15.337315606

S 2.815157877 13.532851424 15.326461230

S -8.314274731 13.500613047 15.346256410

Cu -2.864590000 3.834704000 13.038470000

Cu 0.847998000 3.834704000 13.038470000

Fe2 4.560587000 3.834704000 13.038470000

Fe2 8.273175000 3.834704000 13.038470000

Cu -4.693485000 7.065564000 13.038470000

Fe2 -0.980896000 7.065564000 13.038470000

Fe2 2.731692000 7.065564000 13.038470000

Cu 6.444281000 7.065564000 13.038470000

Fe2 -6.522380000 10.296424000 13.038470000

Fe2 -2.809791000 10.296424000 13.038470000

Cu 0.902797000 10.296424000 13.038470000

Cu 4.615386000 10.296424000 13.038470000

Fe2 -8.351274000 13.527284000 13.038470000

Cu -4.638686000 13.527284000 13.038470000

Cu -0.926097000 13.527284000 13.038470000

Fe2 2.786491000 13.527284000 13.038470000

S 6.416881000 4.884947000 12.301337000

S 2.732171000 4.932763000 12.301337000

S 0.875398000 8.115807000 12.301337000

S -2.809312000 8.163624000 12.301337000

S -4.666085000 11.346667000 12.301337000

S -8.350795000 11.394484000 12.301337000

S -10.207568000 14.577527000 12.301337000

S 0.958076000 14.625344000 12.301337000

S -4.748763000 4.901201000 12.256368000

S -1.008296000 4.949017000 12.256368000

S 4.560108000 8.132061000 12.256368000

S -6.549779000 8.179878000 12.256368000

S -0.981375000 11.362921000 12.256368000

S 2.759092000 11.410738000 12.256368000

S -6.522859000 14.593781000 12.256368000

S -2.782391000 14.641598000 12.256368000

Cu -2.919390000 1.702097000 10.000000000

Fe1 0.793199000 1.702097000 10.000000000

Fe1 4.505787000 1.702097000 10.000000000

Cu 8.218376000 1.702097000 10.000000000

Fe1 -4.748284000 4.932957000 10.000000000

Fe1 -1.035696000 4.932957000 10.000000000

Cu 2.676893000 4.932957000 10.000000000

Cu 6.389481000 4.932957000 10.000000000

Fe1 -6.577179000 8.163817000 10.000000000

Cu -2.864590000 8.163817000 10.000000000

Cu 0.847998000 8.163817000 10.000000000

Fe1 4.560587000 8.163817000 10.000000000

Cu -8.406073000 11.394677000 10.000000000

Cu -4.693485000 11.394677000 10.000000000

Fe1 -0.980896000 11.394677000 10.000000000

Fe1 2.731692000 11.394677000 10.000000000

---------

Structure of (112)-S surface of chalcopyrite (S-Cu type) with H_2_O and •OH before H• migration

O -3.414725197 9.835010615 20.665239741

H -2.819427176 10.156337010 21.371412592

O -6.780202668 7.948695316 24.856209629

H -6.673317888 7.885359589 23.886727335

H -7.732105335 7.800048169 24.997813998

S -1.948474392 4.274872590 21.325920884

S -3.974862901 5.181245559 21.338146252

S -7.454351173 7.461554240 21.284095047

S 4.694266250 9.242598120 21.308679325

S 2.771615352 10.377496902 21.320613620

S -0.601956423 12.280680682 21.359468321

S -3.742949511 14.258976669 21.351408730

S -5.690850839 15.270054985 21.330784805

S 2.146465165 4.316162528 21.343434255

S 6.947264468 5.489980586 21.388426152

S -3.614388085 7.151797256 21.531290492

S 1.452217493 8.811729120 21.373951760

S -8.738421001 10.712889248 21.414946666

S -4.341540627 12.359426962 21.657909736

S 0.695415228 13.822457740 21.394121227

S -9.652507797 15.198770007 21.433462642

Cu -4.626629070 4.893994039 19.093842933

Cu -1.138475724 4.889559688 19.148061631

Fe2 2.628639027 4.889071857 19.163881378

Fe2 6.502994639 4.926405009 19.234632452

Cu -6.665301324 8.012598213 19.208626500

Fe2 -3.057714726 8.247382265 19.468411842

Fe2 0.913222861 8.268103552 19.203350707

Cu 4.628996373 8.231372701 19.146678202

Fe2 -8.338975189 11.302926094 19.227347104

Fe2 -4.435125525 11.197536294 19.580953930

Cu -0.880403009 11.466535491 19.246631857

Cu 2.808647082 11.260693171 19.130054873

Fe2 -10.083369636 14.654685732 19.239784737

Cu -6.379474209 14.609339720 19.134786784

Cu -2.875345580 14.557768188 19.145279381

Fe2 0.928100328 14.455374450 19.228771307

S 4.571826594 6.031147881 18.561010205

S 0.737907358 6.047427065 18.516320263

S -1.056836358 9.269275588 18.555066656

S -4.894576812 9.165712767 18.361008854

S -6.370159557 12.385506247 18.654251417

S -10.203576674 12.452984541 18.509106307

S -12.059323119 15.633318970 18.518141814

S -0.952758162 15.634935550 18.555211302

S -6.506170538 5.893931642 18.299447729

S -2.853784814 6.180410845 18.360684717

S 2.753267795 9.147786695 18.209074875

S -8.368467778 9.237412895 18.261232030

S -2.806355737 12.399251120 18.406429145

S 1.013248844 12.383103517 18.296225049

S -8.236546341 15.672076166 18.346795028

S -4.585372847 15.745713888 18.221391773

Cu -4.721975737 2.752705415 16.206026763

Fe1 -0.984850853 2.738466919 16.082923828

Fe1 2.743225898 2.757531780 15.988328721

Cu 6.403957152 2.740641585 16.234990399

Fe1 -2.806078294 6.014803327 16.109194749

Cu 0.839530656 6.009354228 16.212821152

Cu 4.578850872 5.985414071 16.246823750

Fe1 -6.534933791 5.933630953 16.047140341

Cu -4.722665460 9.223132070 16.098669511

Cu -0.999875590 9.228017914 16.254525711

Fe1 2.761412061 9.170141275 15.978757385

Fe1 -8.365267487 9.207128596 16.020582018

Cu -10.196132621 12.468971140 16.196421603

Cu -6.480096329 12.459919193 16.322276414

Fe1 -2.775911197 12.413359454 16.146022051

Fe1 0.959257562 12.429217294 16.047494529

S 0.851178995 3.889585428 15.313547919

S -2.882786649 3.831958680 15.345674549

S -4.689574003 7.080374313 15.303658064

S 6.415476630 7.022344371 15.321124613

S 4.614411923 10.347751035 15.310491220

S 0.841444362 10.256193252 15.316551064

S -0.932561642 13.546160289 15.338410706

S -4.659596748 13.481509815 15.334715641

S 4.609055853 3.860364386 15.321910691

S 8.264916508 3.779367966 15.329294578

S -0.973683797 7.108274454 15.327494188

S 2.728674419 7.002267223 15.319246821

S -6.542464076 10.360233787 15.312415660

S -2.831808142 10.283230117 15.336089209

S 2.811932197 13.537636646 15.325004358

S -8.316354718 13.504752800 15.347231298

Cu -2.864590000 3.834704000 13.038470000

Cu 0.847998000 3.834704000 13.038470000

Fe2 4.560587000 3.834704000 13.038470000

Fe2 8.273175000 3.834704000 13.038470000

Cu -4.693485000 7.065564000 13.038470000

Fe2 -0.980896000 7.065564000 13.038470000

Fe2 2.731692000 7.065564000 13.038470000

Cu 6.444281000 7.065564000 13.038470000

Fe2 -6.522380000 10.296424000 13.038470000

Fe2 -2.809791000 10.296424000 13.038470000

Cu 0.902797000 10.296424000 13.038470000

Cu 4.615386000 10.296424000 13.038470000

Fe2 -8.351274000 13.527284000 13.038470000

Cu -4.638686000 13.527284000 13.038470000

Cu -0.926097000 13.527284000 13.038470000

Fe2 2.786491000 13.527284000 13.038470000

S 6.416881000 4.884947000 12.301337000

S 2.732171000 4.932763000 12.301337000

S 0.875398000 8.115807000 12.301337000

S -2.809312000 8.163624000 12.301337000

S -4.666085000 11.346667000 12.301337000

S -8.350795000 11.394484000 12.301337000

S -10.207568000 14.577527000 12.301337000

S 0.958076000 14.625344000 12.301337000

S -4.748763000 4.901201000 12.256368000

S -1.008296000 4.949017000 12.256368000

S 4.560108000 8.132061000 12.256368000

S -6.549779000 8.179878000 12.256368000

S -0.981375000 11.362921000 12.256368000

S 2.759092000 11.410738000 12.256368000

S -6.522859000 14.593781000 12.256368000

S -2.782391000 14.641598000 12.256368000

Cu -2.919390000 1.702097000 10.000000000

Fe1 0.793199000 1.702097000 10.000000000

Fe1 4.505787000 1.702097000 10.000000000

Cu 8.218376000 1.702097000 10.000000000

Fe1 -4.748284000 4.932957000 10.000000000

Fe1 -1.035696000 4.932957000 10.000000000

Cu 2.676893000 4.932957000 10.000000000

Cu 6.389481000 4.932957000 10.000000000

Fe1 -6.577179000 8.163817000 10.000000000

Cu -2.864590000 8.163817000 10.000000000

Cu 0.847998000 8.163817000 10.000000000

Fe1 4.560587000 8.163817000 10.000000000

Cu -8.406073000 11.394677000 10.000000000

Cu -4.693485000 11.394677000 10.000000000

Fe1 -0.980896000 11.394677000 10.000000000

Fe1 2.731692000 11.394677000 10.000000000

---------

Structure of the (112)-S surface of chalcopyrite (S-Fe type) with H_2_O and •OH after H• transfer

O -4.778917467 10.663580016 21.215715544

H -4.324201674 11.221434335 21.890644715

H -4.633682687 9.716399913 21.436446379

O -2.669813255 13.148490718 23.930992042

H -1.771948227 13.519926455 24.081617602

S -1.976393800 4.394908126 21.335968884

S -3.912595265 5.494991659 21.337453347

S -7.285102073 7.430226155 21.332622281

S 4.633654765 9.249736311 21.322085091

S 2.683826168 10.350499432 21.332544206

S -0.008700333 11.864501157 21.421982958

S -3.416057450 14.727854841 21.508538573

S -5.701941797 15.273662687 21.443542775

S 1.988869785 4.349437598 21.372165400

S 6.972578882 5.496937850 21.398027935

S -3.146528324 7.412031744 21.343346392

S 1.497670412 8.665461805 21.370900534

S -8.798274670 10.720774534 21.430287670

S -2.857685440 12.960554242 22.281849980

S 0.753394111 13.750001110 21.405193634

S -9.787058841 15.346463051 21.484296811

Cu -4.645608383 4.947004350 19.166634178

Cu -1.082035582 4.840329905 19.170398038

Fe2 2.627776725 4.870192229 19.203117016

Fe2 6.507976941 4.947493740 19.247219486

Cu -6.646800299 7.993525275 19.225391088

Fe2 -2.856696324 8.002504964 19.137854651

Fe2 0.958739299 8.208785104 19.194813952

Cu 4.623867501 8.233920409 19.169178140

Fe2 -8.424895330 11.297274965 19.241539402

Fe2 -4.654534434 11.218794191 19.168145582

Cu -0.802218939 11.398643591 19.328531652

Cu 2.765796912 11.252244707 19.127115811

Fe2 -10.117015303 14.696803294 19.287385469

Cu -6.338309615 14.638082546 19.281146564

Cu -2.815932345 14.551229054 19.226192710

Fe2 0.921785848 14.459834280 19.244533567

S 4.564061105 6.027784969 18.584362635

S 0.756222056 6.009659238 18.485580319

S -0.996940751 9.251478755 18.539321014

S -4.775016000 9.051790297 18.413204660

S -6.516282104 12.465395907 18.602502203

S -10.265292623 12.487233786 18.538658342

S -12.080587723 15.682392371 18.551443518

S -0.934248811 15.657687145 18.553738814

S -6.512232267 5.893046606 18.284207135

S -2.811055196 5.954612496 18.190811453

S 2.762819729 9.150480889 18.197756606

S -8.349543614 9.220642365 18.298806080

S -2.756389674 12.363997493 18.545397705

S 0.996212778 12.415068268 18.261277065

S -8.212324537 15.618402949 18.395032803

S -4.550303372 15.708121066 18.282706061

Cu -4.728886416 2.742496847 16.235742594

Fe1 -0.961385399 2.712234979 16.128244187

Fe1 2.755366131 2.737297519 16.038045725

Cu 6.403613921 2.741528144 16.230806395

Fe1 -2.785180374 5.949137606 15.961772284

Cu 0.855931954 5.984073251 16.185978481

Cu 4.579667648 5.981734125 16.263200375

Fe1 -6.534206432 5.926429787 16.036314950

Cu -4.693022603 9.202020804 16.123639542

Cu -0.985600514 9.237580823 16.227503223

Fe1 2.781182212 9.176074700 15.969082418

Fe1 -8.345094416 9.200073025 16.051925498

Cu -10.201653571 12.466533969 16.227210498

Cu -6.490894490 12.461070504 16.271451499

Fe1 -2.766146782 12.410886686 16.231268406

Fe1 0.956688448 12.444323033 16.020563393

S 0.866668447 3.855404761 15.321693761

S -2.870183223 3.750930091 15.326358117

S -4.683322863 7.066221883 15.296034979

S 6.414643632 7.021605671 15.327100308

S 4.643813850 10.347740994 15.318586071

S 0.861452275 10.263764533 15.312162581

S -0.951984332 13.543098629 15.343611715

S -4.635712945 13.470068490 15.347785323

S 4.601151194 3.858040124 15.329442597

S 8.270190385 3.770695867 15.329185055

S -0.946521917 7.107669660 15.314131253

S 2.738663479 7.004873567 15.318319905

S -6.515307383 10.335806742 15.324842702

S -2.823333904 10.298888694 15.345895952

S 2.819825449 13.539222832 15.323660539

S -8.330888399 13.499347809 15.340405773

Cu -2.864590000 3.834704000 13.038470000

Cu 0.847998000 3.834704000 13.038470000

Fe2 4.560587000 3.834704000 13.038470000

Fe2 8.273175000 3.834704000 13.038470000

Cu -4.693485000 7.065564000 13.038470000

Fe2 -0.980896000 7.065564000 13.038470000

Fe2 2.731692000 7.065564000 13.038470000

Cu 6.444281000 7.065564000 13.038470000

Fe2 -6.522380000 10.296424000 13.038470000

Fe2 -2.809791000 10.296424000 13.038470000

Cu 0.902797000 10.296424000 13.038470000

Cu 4.615386000 10.296424000 13.038470000

Fe2 -8.351274000 13.527284000 13.038470000

Cu -4.638686000 13.527284000 13.038470000

Cu -0.926097000 13.527284000 13.038470000

Fe2 2.786491000 13.527284000 13.038470000

S 6.416881000 4.884947000 12.301337000

S 2.732171000 4.932763000 12.301337000

S 0.875398000 8.115807000 12.301337000

S -2.809312000 8.163624000 12.301337000

S -4.666085000 11.346667000 12.301337000

S -8.350795000 11.394484000 12.301337000

S -10.207568000 14.577527000 12.301337000

S 0.958076000 14.625344000 12.301337000

S -4.748763000 4.901201000 12.256368000

S -1.008296000 4.949017000 12.256368000

S 4.560108000 8.132061000 12.256368000

S -6.549779000 8.179878000 12.256368000

S -0.981375000 11.362921000 12.256368000

S 2.759092000 11.410738000 12.256368000

S -6.522859000 14.593781000 12.256368000

S -2.782391000 14.641598000 12.256368000

Cu -2.919390000 1.702097000 10.000000000

Fe1 0.793199000 1.702097000 10.000000000

Fe1 4.505787000 1.702097000 10.000000000

Cu 8.218376000 1.702097000 10.000000000

Fe1 -4.748284000 4.932957000 10.000000000

Fe1 -1.035696000 4.932957000 10.000000000

Cu 2.676893000 4.932957000 10.000000000

Cu 6.389481000 4.932957000 10.000000000

Fe1 -6.577179000 8.163817000 10.000000000

Cu -2.864590000 8.163817000 10.000000000

Cu 0.847998000 8.163817000 10.000000000

Fe1 4.560587000 8.163817000 10.000000000

Cu -8.406073000 11.394677000 10.000000000

Cu -4.693485000 11.394677000 10.000000000

Fe1 -0.980896000 11.394677000 10.000000000

Fe1 2.731692000 11.394677000 10.000000000

---------

Structure of the (112)-S surface of chalcopyrite (S-Cu type) with H_2_O and •OH after H• transfer

O -2.891521129 9.670158416 22.978075046

H -2.169550071 10.329265014 23.056270840

H -3.211637046 9.509187788 23.882784394

O -0.531649203 11.301333748 22.512257301

H 0.084310084 10.577431492 22.177427041

S -1.983297691 4.278152886 21.313287151

S -3.957034644 5.273454312 21.289789730

S -7.436192892 7.464195314 21.331290529

S 4.718195696 9.239261983 21.320598587

S 2.871764017 10.467489739 21.321489793

S -0.761901205 12.440241836 21.317200777

S -3.759846105 14.042056833 21.311312144

S -5.679346493 15.134650660 21.322436510

S 2.296993935 4.145760174 21.393709344

S 6.919495702 5.506721738 21.392288639

S -3.365714739 7.216485939 21.450843527

S 1.410412656 9.012329832 21.428023617

S -8.724649828 10.725734086 21.415301123

S -4.317386719 12.082990062 21.442245812

S 0.836811077 13.736539377 21.438821253

S -9.712767313 15.205382479 21.424231823

Cu -4.661151439 4.890723327 19.087721971

Cu -1.088337928 4.869107012 19.169104015

Fe2 2.657262494 4.833436442 19.218674952

Fe2 6.497907099 4.947062286 19.234233201

Cu -6.637853103 8.053266086 19.274580444

Fe2 -2.955670453 7.998282739 19.299630962

Fe2 0.914013874 8.276370535 19.261365884

Cu 4.632442426 8.236604358 19.158696772

Fe2 -8.392465521 11.294965583 19.217607683

Fe2 -4.614276248 11.437491236 19.255149201

Cu -0.893258294 11.424825719 19.176418272

Cu 2.811747370 11.279678501 19.123063397

Fe2 -10.115705598 14.661296789 19.228237967

Cu -6.404397157 14.665767299 19.132023702

Cu -2.856066049 14.546073652 19.156062029

Fe2 0.917630883 14.425492830 19.264767354

S 4.561144098 6.034492764 18.572229958

S 0.781837239 6.035701955 18.581084834

S -1.084261559 9.233988721 18.597186031

S -4.783557311 9.215656191 18.577267355

S -6.530282844 12.484435659 18.516020107

S -10.224824908 12.472438354 18.484463082

S -12.083944347 15.627794359 18.514905896

S -0.948024089 15.649896237 18.570406494

S -6.514197199 5.941442427 18.316600125

S -2.830406730 6.000434164 18.225618726

S 2.751310998 9.152951683 18.242246190

S -8.347848701 9.224352001 18.276899850

S -2.770472109 12.415876455 18.318142543

S 0.993551861 12.360455459 18.293011017

S -8.269780261 15.668099454 18.328624494

S -4.569616461 15.721410316 18.215000324

Cu -4.734753142 2.743440936 16.209487767

Fe1 -0.974391413 2.738048116 16.071189768

Fe1 2.747944241 2.766403249 15.981667949

Cu 6.409140742 2.762956905 16.251687152

Fe1 -2.802922923 5.986678813 15.990616646

Cu 0.860020604 6.009201179 16.265280129

Cu 4.580259640 5.993613110 16.253283488

Fe1 -6.521587374 5.953177093 16.063100598

Cu -4.691444824 9.230617688 16.259197785

Cu -0.993907882 9.215055675 16.272988048

Fe1 2.767537125 9.171146455 16.009063232

Fe1 -8.340557934 9.206366020 16.036954191

Cu -10.211594446 12.454892342 16.184405356

Cu -6.504807018 12.459633991 16.208886357

Fe1 -2.758733439 12.413430318 16.063075087

Fe1 0.952274124 12.418406064 16.041554990

S 0.856901057 3.902891398 15.319386766

S -2.888984761 3.789307718 15.321490793

S -4.690668551 7.123866619 15.319815868

S 6.421266658 7.024721077 15.325737400

S 4.629757444 10.329207641 15.316000008

S 0.844588565 10.240992128 15.320653442

S -0.913910502 13.573994278 15.327709073

S -4.657093523 13.493637375 15.316149718

S 4.613513427 3.869526742 15.321873532

S 8.269513318 3.799884066 15.333201276

S -0.942375402 7.098432734 15.326577225

S 2.729781732 7.008346836 15.328316486

S -6.492833031 10.322910288 15.326508990

S -2.826868660 10.258428160 15.339110401

S 2.802260448 13.532126378 15.321401465

S -8.348664473 13.511244483 15.326133271

Cu -2.864590000 3.834704000 13.038470000

Cu 0.847998000 3.834704000 13.038470000

Fe2 4.560587000 3.834704000 13.038470000

Fe2 8.273175000 3.834704000 13.038470000

Cu -4.693485000 7.065564000 13.038470000

Fe2 -0.980896000 7.065564000 13.038470000

Fe2 2.731692000 7.065564000 13.038470000

Cu 6.444281000 7.065564000 13.038470000

Fe2 -6.522380000 10.296424000 13.038470000

Fe2 -2.809791000 10.296424000 13.038470000

Cu 0.902797000 10.296424000 13.038470000

Cu 4.615386000 10.296424000 13.038470000

Fe2 -8.351274000 13.527284000 13.038470000

Cu -4.638686000 13.527284000 13.038470000

Cu -0.926097000 13.527284000 13.038470000

Fe2 2.786491000 13.527284000 13.038470000

S 6.416881000 4.884947000 12.301337000

S 2.732171000 4.932763000 12.301337000

S 0.875398000 8.115807000 12.301337000

S -2.809312000 8.163624000 12.301337000

S -4.666085000 11.346667000 12.301337000

S -8.350795000 11.394484000 12.301337000

S -10.207568000 14.577527000 12.301337000

S 0.958076000 14.625344000 12.301337000

S -4.748763000 4.901201000 12.256368000

S -1.008296000 4.949017000 12.256368000

S 4.560108000 8.132061000 12.256368000

S -6.549779000 8.179878000 12.256368000

S -0.981375000 11.362921000 12.256368000

S 2.759092000 11.410738000 12.256368000

S -6.522859000 14.593781000 12.256368000

S -2.782391000 14.641598000 12.256368000

Cu -2.919390000 1.702097000 10.000000000

Fe1 0.793199000 1.702097000 10.000000000

Fe1 4.505787000 1.702097000 10.000000000

Cu 8.218376000 1.702097000 10.000000000

Fe1 -4.748284000 4.932957000 10.000000000

Fe1 -1.035696000 4.932957000 10.000000000

Cu 2.676893000 4.932957000 10.000000000

Cu 6.389481000 4.932957000 10.000000000

Fe1 -6.577179000 8.163817000 10.000000000

Cu -2.864590000 8.163817000 10.000000000

Cu 0.847998000 8.163817000 10.000000000

Fe1 4.560587000 8.163817000 10.000000000

Cu -8.406073000 11.394677000 10.000000000

Cu -4.693485000 11.394677000 10.000000000

Fe1 -0.980896000 11.394677000 10.000000000

Fe1 2.731692000 11.394677000 10.000000000
